# Elimination of *Pseudomonas aeruginosa* through Efferocytosis upon Binding to Apoptotic Cells

**DOI:** 10.1371/journal.ppat.1006068

**Published:** 2016-12-15

**Authors:** Darío Capasso, María Victoria Pepe, Jéssica Rossello, Paola Lepanto, Paula Arias, Valentina Salzman, Arlinet Kierbel

**Affiliations:** 1 Instituto de Investigaciones Biotecnológicas Dr. Rodolfo A. Ugalde (IIB-INTECH), Universidad Nacional de San Martín, Consejo Nacional de Investigaciones Científicas y Técnicas (UNSAM-CONICET), San Martín, Buenos Aires, Argentina; 2 Institut Pasteur de Montevideo, Montevideo, Uruguay; Ohio State University, UNITED STATES

## Abstract

For opportunistic pathogens such as *Pseudomonas aeruginosa*, the mucosal barrier represents a formidable challenge. Infections develop only in patients with altered epithelial barriers. Here, we showed that *P*. *aeruginosa* interacts with a polarized epithelium, adhering almost exclusively at sites of multi-cellular junctions. In these sites, numerous bacteria attach to an extruded apoptotic cell or apoptotic body. This dead cell tropism is independent of the type of cell death, as *P*. *aeruginosa* also binds to necrotic cells. We further showed that *P*. *aeruginosa* is internalized through efferocytosis, a process in which surrounding epithelial cells engulf and dispose of extruded apoptotic cells. Intracellularly, along with apoptotic cell debris, *P*. *aeruginosa* inhabits an efferocytic phagosome that acquires lysosomal features, and is finally killed. We propose that elimination of *P*. *aeruginosa* through efferocytosis is part of a host defense mechanism. Our findings could be relevant for the study of cystic fibrosis, which is characterized by an exacerbated number of apoptotic cells and ineffective efferocytosis.

## Author Summary

*Pseudomonas aeruginosa* is an opportunistic pathogen that infects vulnerable patients, such as those with cystic fibrosis or hospitalized in intensive care units. An advance towards understanding infections caused by *P*. *aeruginosa* would be to fully elucidate the mechanisms that operate in the bacteria-epithelial barrier interplay. Here, we showed that *P*. *aeruginosa* exhibits a remarkable tropism towards dead cells. As bacteria interact with a polarized epithelium, they attach and aggregate almost exclusively on apoptotic cells extruded from the epithelium, while the rest of the surface seems reluctant to bacterial adhesion. We further showed that *P*. *aeruginosa* is internalized by epithelial cells surrounding the infected apoptotic cell through efferocytosis, a process in which apoptotic cells are engulfed and disposed of by other cells. Bacteria are eliminated intracellularly. Our findings may help to understand why contexts such as cystic fibrosis, where apoptotic cells are unusually produced and efferocytosis fails, favor *P*. *aeruginosa* colonization.

## Introduction

*Pseudomonas aeruginosa* is a ubiquitous environmental Gram-negative bacterium and an opportunistic pathogen. It is the causative agent of both acute and chronic human infections. Acute infections are major problems in immunocompromised patients, burn victims and patients requiring mechanical ventilation. Chronic pulmonary infections with *P*. *aeruginosa* severely impair the quality of life and life expectancy of cystic fibrosis (CF) patients and are a major factor contributing to their mortality [1, 2]. For opportunistic pathogens such as *P*. *aeruginosa*, the mucosal barrier represents a formidable challenge for colonization and bacterial-mediated damage. Thus, infections develop only in patients with altered epithelial cell barriers, including direct trauma, indwelling catheters, or patients receiving cytotoxic chemotherapy. Regarding CF, the sequence of events predisposing to airway infection has been debated for years. A consensus now exists that the respiratory tract pathophysiology in CF largely results from the inability to secrete Cl^−^ and regulate Na^+^ absorption, which causes dehydration and accumulation of hyper-viscous mucus [3]. CF is also characterized by robust airway inflammation and accumulation of apoptotic cells [4, 5].

In healthy individuals, billions of cells die by apoptosis each day. These cells must be efficiently removed to prevent secondary necrosis and the release of pro-inflammatory cell contents. Professional and non-professional phagocytes, such as epithelial cells, engulf and dispose of apoptotic cells in different tissues in a process called “efferocytosis”, from the Latin “to bury” [6–9]. Previous to removal of apoptotic cells, it is also crucial for epithelial tissues to preserve the barrier function as dying cells are expelled out of the epithelium. This is achieved through a process termed “apoptotic cell extrusion”. During extrusion, the apoptotic cell signals its neighbors to reorient and form an actin/myosin contractile ring that squeezes the dying cell out of the monolayer [10, 11]. This contraction not only ejects the dying cell but also closes any gaps that may have resulted from the exit of the dying cell. The extrusion process leaves a characteristic rosette-like arrangement of the surrounding cells with a central multicellular junction that is maintained for several hours after the actual extrusion event takes place [12]. Apoptotic cell extrusion is conserved in all *in vivo* epithelia so far examined, from *Drosophila* to humans, as well as in culture model systems of polarized epithelial cells.

We have recently reported that *P*. *aeruginosa* interacts with cultured polarized epithelial monolayers adhering as aggregates at very localized spots of the apical surface and that the rest of the surface shows resistance to *P*. *aeruginosa* attachment. We have further demonstrated that aggregates are formed *in situ* in the order of minutes and that they can be rapidly internalized into epithelial cells in a Lyn-phosphatidylinositol 3-kinase (PI3K)-dependent manner [13, 14].

About 50% of *P*. *aeruginosa* clinical isolates studied can be measurably internalized into non-phagocytic cells both *in vivo* and *in vitro* [15]. However, the role of internalization in the infection process is not clearly understood and which is the intracellular fate of *P*. *aeruginosa* is not known. Few studies have measured *P*. *aeruginosa* long-term survival inside non-phagocytic cells. Some of them have found that *P*. *aeruginosa* is unable to survive intracellularly long past 24 h, inside endothelial [16] and epithelial cells [17, 18]. On the other hand, García Medina et al. suggested that airway epithelial cells develop pod-like clusters of intracellular *P*. *aeruginosa* that persist for at least 3 days [19]. More recently, it has been described that the Type-Three Secretion System effector ExoS promotes *P*. *aeruginosa* intracellular survival within corneal epithelial cells associated with the formation of membrane bleb niches [20, 21].

In this study, we showed that *P*. *aeruginosa* interaction with the epithelial barrier preferentially occurs at sites of multicellular junctions formed by four or more cells. We further showed that, in those sites, *P*. *aeruginosa* attaches to extruded apoptotic cells or apoptotic bodies. *P*. *aeruginosa* is then internalized by surrounding epithelial cells through efferocytosis. Inside epithelial cells bacteria inhabit, along with apoptotic cell debris, an efferocytic phagosome that acquires lysosomal features. *P*. *aeruginosa* is then eliminated intracellularly after 24 hours.

## Results

### *Pseudomonas aeruginosa* aggregates at sites of multicellular junction

**Fig 1:**
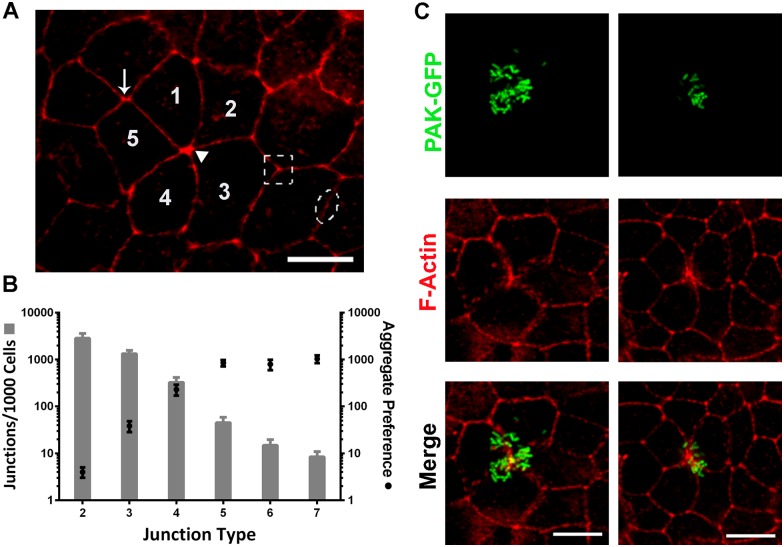
*P*. *aeruginosa* aggregates at multicellular junctions. Cell junction types and *P*. *aeruginosa* adhesion sites were analyzed by quantitative confocal microscopy. (A) Representative micrograph showing different cell junctions types: Junction of five cells (full arrowhead), junction of four cells (arrow), junction of three cells (dotted square) and junction of two cells (dotted ellipse). The numbers indicate the cells that come together to form a junction of five. (B) The gray bars (left, y-axis) represent the frequency of junction types, based on the analysis of four randomly imaged regions (~300 cells/region) of a polarized MDCK monolayer. The black circles (right, y-axis) represent the number of *P*. *aeruginosa* aggregates formed per junction type normalized to the junction type frequency. Data are presented as mean ± SEM. Pearson correlation coefficient r = 0.95, *p<*0.05. (C) Projected confocal Z stacks showing examples of *P*. *aeruginosa* adhered to multicellular junctions formed by four (left) and five cells (right). PAK-GFP: green and F-actin: red. Scale bars: 10 μm.

We have recently shown that *P*. *aeruginosa* adheres to the epithelial barrier by recruitment of free-swimming bacteria to localized spots on the cell surface and that 94.0 ± 1.0% of cell surface-associated bacteria form part of the aggregates [13]. As we have consistently noted that aggregation occurs at cell-cell junctions, in the present study, we carried out a quantitative analysis of *P*. *aeruginosa* adhesion/aggregation tropism. We found that, on average, about 350 bacteria/field (~ 300 epithelial cells) were associated to the surface. We evaluated 10–20 fields per experiment and analyzed at least three independent experiments. The number of bacteria per aggregate was highly variable (from 6 (defined by us as the minimum to form an aggregate) to 300). We carried out an analysis similar to one previously done to characterize *Listeria monocytogenes* attachment to the epithelial barrier [12]. First, the frequency of each “junction type” throughout the monolayer was characterized. The “junction type” was defined according to the number of cells that came together to form a junction (Fig 1A). Then, this was related to sites of *P*. *aeruginosa* aggregation. Polarized MDCK monolayers were infected with *P*. *aeruginosa* strain K expressing GFP (PAK-GFP) for 3 h and fixed. Epithelial cells were stained with phalloidin-rhodamine for F-actin. The number of aggregates associated with each junction type was counted. As shown in Fig 1B, *P*. *aeruginosa* adherence/aggregation happens preferentially on multicellular junctions formed by four or more epithelial cells. Fig 1C shows examples of aggregates on multicellular junctions formed by four (left) or five cells (right).

### *P*. *aeruginosa* adheres to extruded apoptotic cells and apoptotic bodies

**Fig 2:**
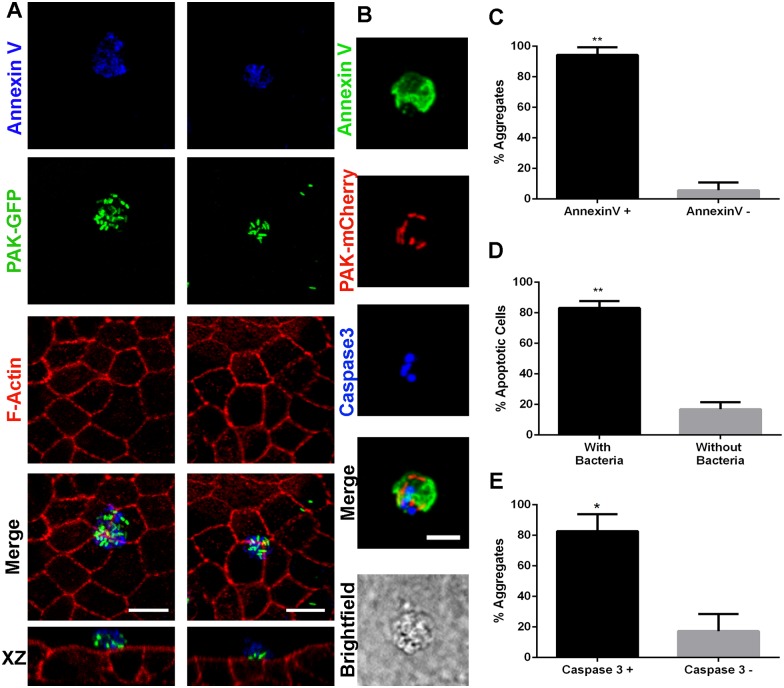
*P*. *aeruginosa* adheres to extruded apoptotic cells. (A) Projected confocal Z stacks and orthogonal sections (lower images) of transwell-grown monolayers infected with PAK. *P*. *aeruginosa* adhered to an extruded apoptotic cell (left) and to an apoptotic body (right). PAK-GFP: green, F-actin: red and Annexin V: blue. (B) Glass-grown monolayers infected with PAK. Apoptotic cells to which *P*. *aeruginosa* adheres have active Caspase 3. PAK-mCherry: red, Annexin V: green and active Caspase-3: blue. Brightfield depicts the monolayer underneath. (C) Percentage of aggregates formed at Annexin V-positive sites. (D) Percentage of Annexin V-positive spots with at least one adhered bacterium. (E) Percentage of aggregates formed at active Caspase 3-positive sites. Data are presented as mean ± SEM. *p<0.05, Student’s *t*-test. Scale bars: 10 μm.

Multicellular junctions can be formed when apoptotic cells are extruded apically from the epithelial monolayer. As we found *P*. *aeruginosa* aggregates at the apical surface, right above multicellular junctions, we wondered whether bacteria could be adhering to extruded apoptotic cells. MDCK monolayers infected with PAK-GFP were labeled with Annexin V-Alexa 647 and then fixed, permeabilized and stained with phalloidin-rhodamine. Annexin V binds specifically to phosphatidylserine, which is exposed to the outer leaflet of the plasma membrane early in the apoptosis pathway [22, 23]. Annexin V staining revealed the presence of apoptotic cells or apoptotic bodies at the sites of *P*. *aeruginosa* attachment. Some apoptotic bodies were small, and would have probably remained unnoticed without Annexin V staining. As shown in Fig 2A, numerous bacteria bound together to an extruded Annexin V-positive cell (left panel) and to an apoptotic body (right panel). Very few bacteria were found attached to living cells at the monolayer. Note that extruded cells to which *P*. *aeruginosa* had attached did not stain for phalloidin. This is a late apoptotic cell feature that has been previously reported [24]. Cells with adhered bacteria were also positive for active caspase-3 staining, confirming their apoptotic status (Fig 2B). To quantify the phenomena observed, fields were systematically scanned and each *P*. *aeruginosa* aggregate was classified as bound to Annexin V-positive or -negative spots or, in a different set of experiments, to sites positive or negative for active caspase-3 staining. We found that 94.3 ± 2.5% of the aggregates were on Annexin V-positive spots (Fig 2C) and that 83.1 ± 2.7% of Annexin V-positive spots had at least one bacterium attached (Fig 2D). Also, we found that 82.7 ± 11.2% of the aggregates were on active caspase-3-positive sites (Fig 2E).

Several reports indicate that *P*. *aeruginosa* is able to induce apoptosis both through direct interaction with the host cells or through secreting factors [25]. Although to reach late stages of apoptosis upon their induction by *P*. *aeruginosa* infection epithelial cells need times longer than those used in our settings [26, 27], we checked whether *P*. *aeruginosa* infection increased the number of apoptotic cells. No difference was found comparing the average of Annexin V-positive spots *per field* between infected and uninfected monolayers (7.4 ± 1.1 and 7.2 ± 0.9 respectively). This result suggests that the cells to which *P*. *aeruginosa* binds were apoptotic before infection.

Two previously characterized *P*. *aeruginosa* cystic fibrosis isolates (one mucoid (isolate 2b) and one non-mucoid (isolate 6) [28]) also showed adhesion to extruded apoptotic cells (S1 Fig), demonstrating that this is not a strain-dependent phenomenon. In addition, PAK apoptotic cell tropism was also observed in the human bronchial epithelial cell line 16HBE14o- (S2 Fig), showing that this is not a cell line-dependent phenomenon either.

### The dead cell tropism of *P*. *aeruginosa* is independent of the type of cell death

**Fig 3:**
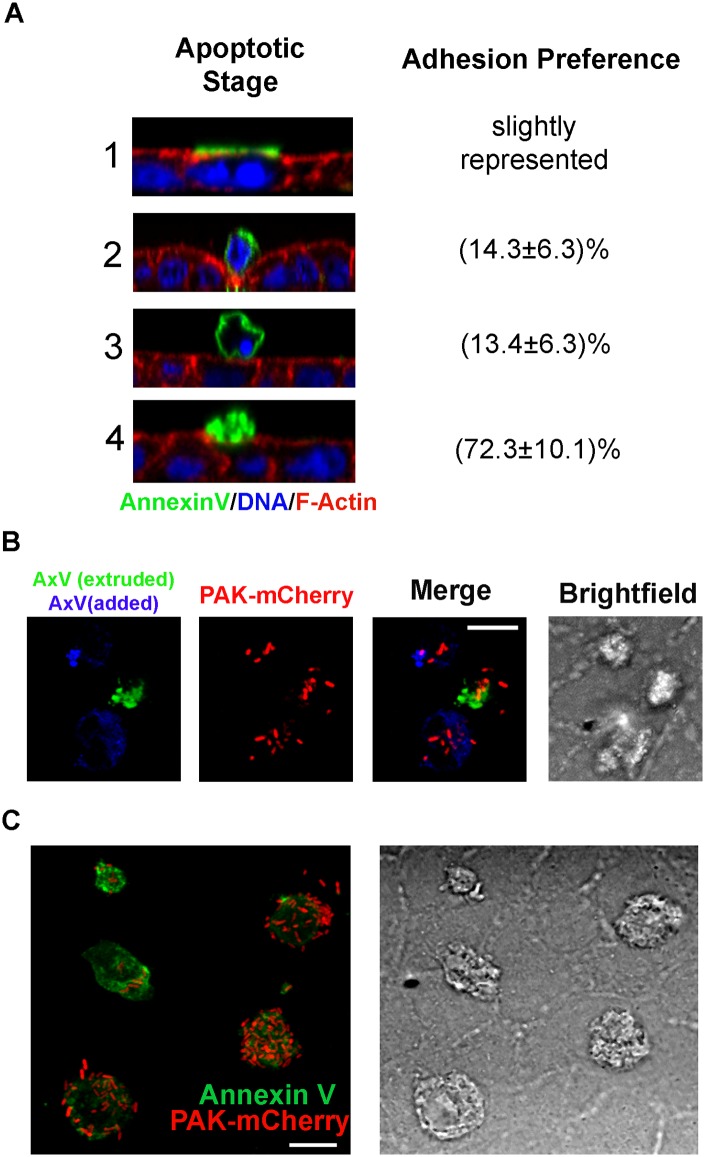
*P*. *aeruginosa* adheres to both apoptotic and necrotic cells. (A) Representative micrographs showing orthogonal sections of apoptotic cell stages and percentage of bacteria adhered to each stage. Data were normalized to the frequency of each apoptotic cell stage. F-actin: red, TO-PRO3 (DNA stain): blue, Annexin V: green. (B) Apoptotic cells extruded from glass-grown MDCK monolayers were stained with Annexin V-Alexa 488 (green). UV-generated apoptotic cells stained with Annexin V-Alexa 647 (blue) were then added to monolayers right before infection with PAK-mCherry (red). (C) H_2_O_2_-generated necrotic cells were stained with Annexin V-Alexa 488 (green) and added to glass-grown MDCK monolayers right before PAK-mCherry infection (red). B and C show projected confocal Z stacks. Scale bars: 10 μm.

To better characterize the phenomenon observed, we arbitrarily defined four stages in the apoptotic cell extrusion process and quantified *P*. *aeruginosa* binding by stage. Cells inserted in the monolayer with positive apical Annexin V staining were classified as stage one. Cells being squeezed out of the monolayer, with an actin ring at the interface with their live neighbors, were classified as stage two. Rounded cells, completely outside the monolayer, with positive Annexin V plasma membrane staining and condensed nucleus were classified as stage three. Finally, cells outside the monolayer and Annexin V-positive, but collapsed and apparently split into apoptotic bodies were classified as stage four. The percentage of *P*. *aeruginosa* binding per stage was calculated (Fig 3A). Most bacteria attached to late apoptotic cells. Next, we generated late apoptotic cells through UV irradiation of trypsin-detached MDCK cells and further incubation for 12 h (S3 Fig, left panel). Cells were stained with Annexin V-Alexa 647 and added to an intact, glass grown MDCK monolayer that had been previously stained with Annexin V-Alexa 488 (to label extruded apoptotic cells). Right after, monolayers were infected with PAK-mCherry. After 3 h, samples were washed with phosphate-buffered saline (PBS) and fixed. *P*. *aeruginosa* attached both to apoptotic cells extruded from the monolayer and to added UV-irradiated cells (Fig 3B). *P*. *aeruginosa* did not attach to trypsin-detached living MDCK cells added to the monolayers (S4 Fig). These results further confirm that *P*. *aeruginosa* adheres to cells that were previously apoptotic and demonstrates that extrusion *per se* is not necessary for *P*. *aeruginosa* attachment.

As late apoptotic cells share some features with necrotic cells, we wondered whether *P*. *aeruginosa* would adhere to this type of dead cells as well. Necrotic cells were generated treating MDCK cells with H_2_O_2_ and further incubation for 20 h (S3 Fig, right panel). Cells were stained with Annexin V and added right before infection with PAK-mCherry to an intact, glass-grown MDCK monolayer. As shown in Fig 3C, *P*. *aeruginosa* adhered to necrotic cells as well. We concluded that the dead cell tropism of *P*. *aeruginosa* is independent of the mechanism by which target cells had died. Binding of *P*. *aeruginosa* to dead cells was assayed on polarized monolayers, since, as mentioned, binding to the apical surface of polarized living cells is very low. Instead, when binding of *P*. *aeruginosa* to dead cells was assayed directly on glass or plastic, background binding was very high.

### *P*. *aeruginosa* is internalized by epithelial cells through efferocytosis

**Fig 4:**
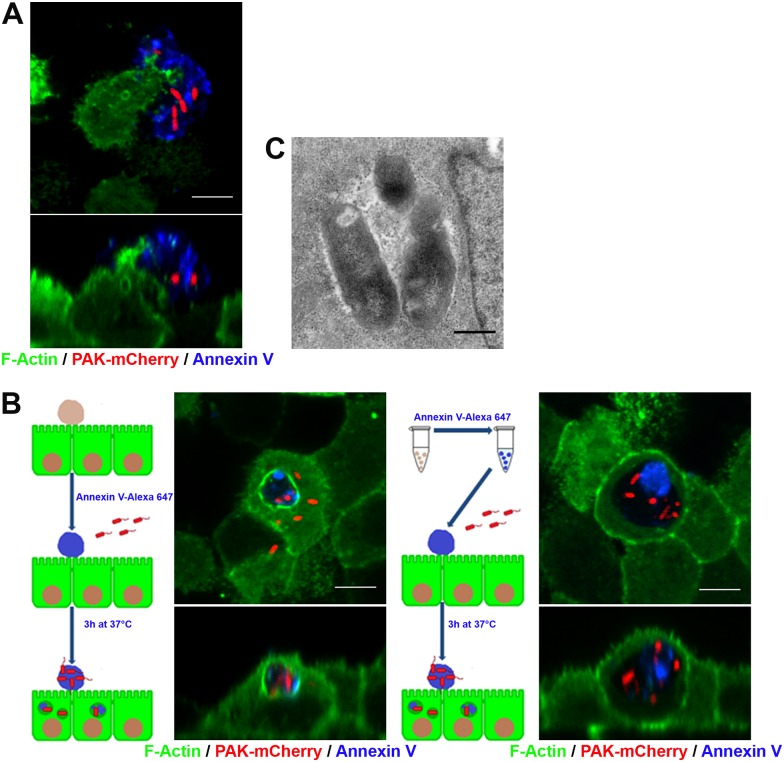
*P*. *aeruginosa* is internalized through efferocytosis. Lifeact-GFP MDCK monolayers (green) were stained with Annexin V-Alexa 647 (blue), infected with PAK-mCherry (red) and incubated for 3 h (A and left micrograph in B). Alternatively, UV-irradiated Annexin V-stained wt MDCK cells were added to lifeact-GFP MDCK monolayers followed by PAK-mCherry infection and incubation for 3 h (B, right micrograph). Scale bars: 5 μm. (A) Top view and orthogonal section of a membrane protrusion extending over an apoptotic cell with adhered bacteria. (B) Schemes describing the strategies and corresponding confocal xy planes (upper images) and orthogonal views (lower images) of efferocytic vesicles containing both apoptotic cell debris and bacteria. (C) Transmission electron microscopy of an intracellular membrane vesicle containing several bacteria. Wt MDCK cells monolayers were infected with wt PAK for 3 h. Scale bar: 500 nm.

As mentioned, the extrusion process leaves a characteristic rosette-like arrangement of the surrounding cells with a central multicellular junction. We consistently observed membrane protrusions coming out of these surrounding cells and extending over extruded apoptotic cells (Fig 4A). We also consistently found numerous bacteria inside these surrounding cells. The percentage of internalized bacteria related to total cell-associated bacteria after 3 hours of infection was variable, typically ranging between 15 and 50%. We wondered whether *P*. *aeruginosa* internalization could be associated with efferocytosis of apoptotic cells or apoptotic bodies by neighboring epithelial cells. Thus, first, we checked whether efferocytosis occurred in our cell culture system. Transwell grown lifeact-GFP MDCK monolayers were stained with Annexin V in binding buffer for 15 min (to stain extruded apoptotic cells) and incubated back in serum-free minimum essential medium (MEM) at 37°C for 3 h. Alternatively, apoptotic cells from overgrown monolayers or generated by UV irradiation were stained with Annexin V-Alexa, added to lifeact-GFP MDCK monolayers and incubated in MEM at 37°C for 3 h. Samples were then fixed and analyzed by confocal microscopy. In all cases, Annexin V-positive cell debris was found within intracellular vesicles, showing that efferocytosis had taken place (S5 Fig). Next, experiments following those protocols, but including infection with PAK-mCherry, were performed. In the first case, infection was done right after monolayers were incubated back in MEM. In the second case, monolayers were infected right after addition of apoptotic cells. Schemes describing those strategies are shown in Fig 4B. Independently of the protocol, intracellular vesicles containing both apoptotic cell debris and bacteria were found (Fig 4B). We found that 59.7 ± 7.0% of internalized bacteria were inside cells that had intracellular apoptotic cell debris as well. The same phenomenon was observed in MDCK cells infected with cystic fibrosis isolates 2b and 6 (S6 Fig) and with 16HBE14o- cells infected with PAK (S7 Fig). Note that bacteria-containing vesicles are, as previously described for the efferocytic phagosome, vacuous, with fluid filling the space between bacteria and/or the apoptotic cell membrane and phagosome membrane [29]. Also, we noticed that some efferosomes were surrounded by F-actin. Transmission electron microscopy of an intracellular membrane vesicle containing several bacteria is shown in Fig 4C.

### Inhibition of efferocytosis reduces *P*. *aeruginosa* internalization

**Fig 5:**
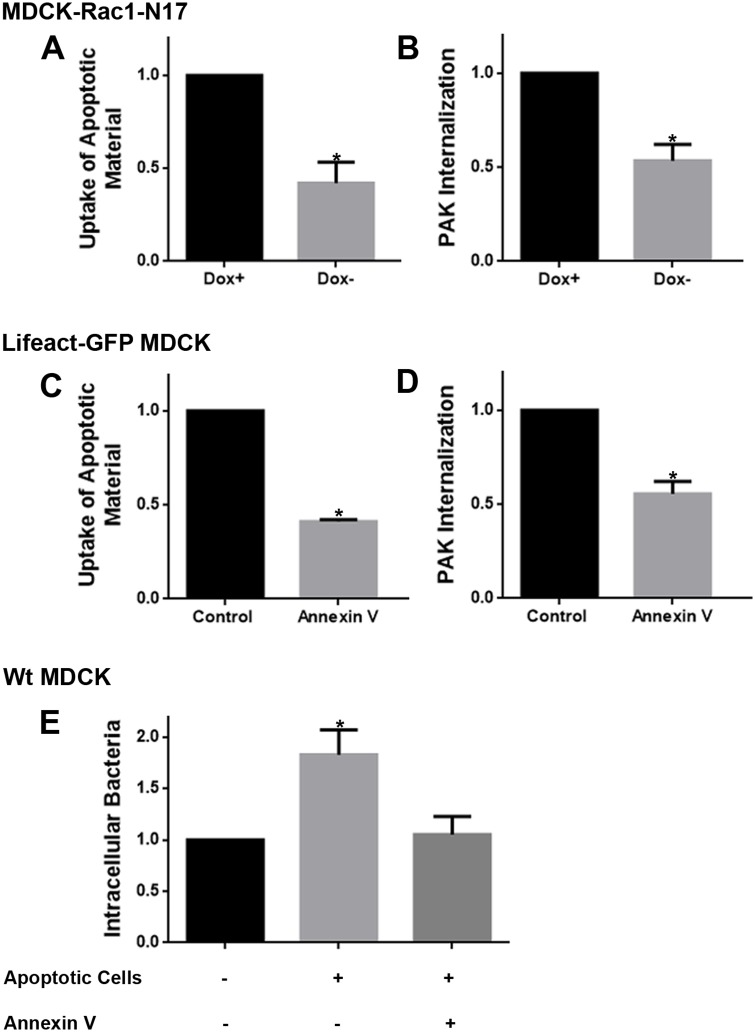
Expression of dominant negative Rac1 and preincubation with unlabeled Annexin V inhibit *P*. *aeruginosa* internalization. (A) Proportion of internalized apoptotic material (Intracellular/Total cell-associated apoptotic material). (B) Proportion of internalized *P*. *aeruginosa* (Internalized/Total cell-associated bacteria). (A and B) Apoptotic cells generated by UV irradiation were stained with Annexin V-Alexa 647 and added to MDCK-Rac1-N17 filter-grown monolayers and infected with PAK-GFP. The cell-associated apoptotic material and cell-associated bacteria were quantified by image analysis as described in M&M. Dox^+^ (control) Dox^-^ (dominant negative Rac1). Data were normalized to control. (C) Proportion of internalized apoptotic material after pre-incubating CellTrace-labeled apoptotic cells with unlabeled Annexin V or with binding buffer alone (control) and adding them to transwell-grown lifeact-GFP MDCK monolayers. (D) Proportion of internalized *P*. *aeruginosa* after pre-incubating transwell-grown lifeact-GFP MDCK monolayers with unlabeled Annexin V for 15 min in binding buffer. (E) Transwell-grown MDCK monolayers were infected with *P*. *aeruginosa* and internalization was measured by standard antibiotic protection assays. When indicated, apoptotic cells from overgrown cultures, pre-incubated with binding buffer alone or with unlabeled-Annexin V, were added to monolayers right before infection. The mean of colony forming units (CFUs) ± SEM was calculated. Data were normalized to control. *p<0.05 vs. control, one-sample *t*-test.

We next evaluated whether inhibition of efferocytosis decreased *P*. *aeruginosa* internalization. The small GTPase Rac1 has been shown to be a key regulator of apoptotic cell engulfment [30–32]. Thus, we used a MDCK cell line that conditionally expressed a dominant-negative (N17) Myc-tagged version of Rac1 under the control of the tetracycline-repressible transactivator [33]. Monolayers expressed exogenous Rac1 in the absence of doxycycline, but not in the presence of 20 ng ml^-1^ doxycycline. Apoptotic cells generated by UV irradiation were stained with Annexin V-Alexa 647 and added to MDCK-Rac1-N17 filter-grown monolayers followed by infection with PAK-GFP for 3 h as described above. After fixation, epithelial cells were stained with phalloidin-rhodamine. Monolayer-associated bacteria and monolayer-associated apoptotic cells/bodies/debris (i.e. apoptotic material) were quantified using the 3D-Object Counter plugin for ImageJ as detailed in the M&M section. Extracellular or intracellular localization was determined visually. Fig 5A shows the proportion of intracellular apoptotic material relative to total monolayer-associated apoptotic material. As expected, Rac1-N17 cells showed impaired uptake of apoptotic cells.

Remarkably, internalization of *P*. *aeruginosa* was also decreased in Rac1-N17 cells (Fig 5B). These results suggest that efferocytosis is important for internalization of *P*. *aeruginosa* into epithelial cells.

To further confirm this, efferocytosis was inhibited by shielding the phosphatidylserine exposed on the cell surface of apoptotic cells with recombinant, unlabeled Annexin V. First, we evaluated whether inhibition of efferocytosis by Annexin V occurred in our epithelial cell system. MDCK cells were labeled with CellTrace (ThermoFisher), a reagent that diffuses into cells and binds covalently to intracellular amines, rendering a stable, well-retained fluorescent staining. Then, the cells were made apoptotic through UV irradiation as described, and incubated with unlabeled Annexin V (15 microgram/10^5^ cells) or in binding buffer (BB) alone (control) for 15 min. It is worth noting that the concentration of Annexin V used to block efferocytosis is approximately seventy-fold higher than the concentration of Alexa-AnnexinV used to label dead cells. Apoptotic cells were added to transwell-grown MDCK monolayers for 3 h and samples were washed and fixed. As mentioned above, efferocytosis was evaluated using the 3D-Object Counter plugin for ImageJ (an example of such procedure is shown in S8 Fig). Pre-incubation with Annexin V reduced the uptake of apoptotic material to 41.00 ± 0.90% (Fig 5C).

We then evaluated whether pre-incubation with Annexin V also inhibited *P*. *aeruginosa* internalization. Transwell-grown MDCK monolayers were pre-incubated with unlabeled Annexin V or BB alone (control) and then infected with *P*. *aeruginosa* for 3 h. In this case, intra- or extracellular bacteria were distinguished through staining with an anti-*Pseudomonas* antibody and image analysis, as described in [34]. Unlabeled Annexin V treatment not only protected the cells from Annexin V-Alexa 488 staining but also led to a significant reduction of intracellular bacteria (Fig 5D), while total-associated bacteria was not significantly changed (S9 Fig). Following a different approach, apoptotic cells from overgrown cultures were added to MDCK monolayers followed by *P*. *aeruginosa* infection (multiplicity of infection (MOI) = 10). Internalization was evaluated through standard antibiotic protection assays. Addition of apoptotic cells significantly increased *P*. *aeruginosa* internalization. Moreover, such increase was prevented by pre-incubating apoptotic cells with unlabeled Annexin V (Fig 5E).

Taken together, our results demonstrate that *P*. *aeruginosa* is internalized into epithelial cells through efferocytosis.

### *P*. *aeruginosa* is eliminated inside epithelial cells

**Fig 6:**
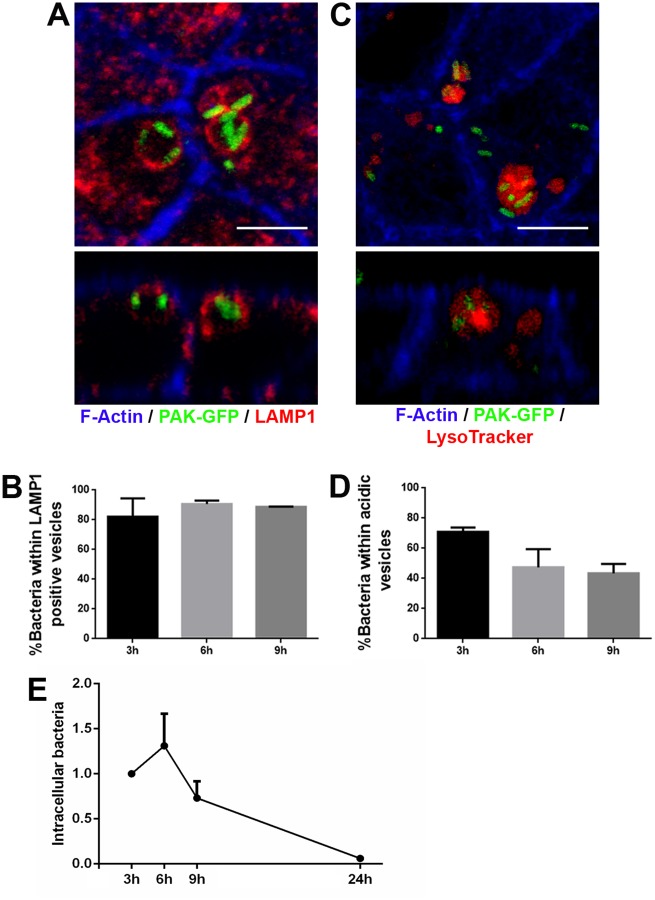
*P*. *aeruginosa* efferocytic phagosome acquires lysosomal features. (A) Projected confocal Z stack (top) and orthogonal section (bottom) showing LAMP1-positive vesicles containing bacteria. F-actin: blue, PAK-GFP: green and LAMP1: red. (B) Percentage of intracellular *P*. *aeruginosa* found within LAMP1 vesicles over time. (C) Projected confocal Z stack (top) and orthogonal section (bottom) showing acidic vesicles containing bacteria. F-actin: blue, PAK-GFP: green and LysoTracker: red. (D) Percentage of intracellular *P*. *aeruginosa* found within acidic vesicles over time. (E) Intracellular *P*. *aeruginosa* survival over time. The mean of CFUs ± SEM was calculated. Data were normalized to time 3 h. Scale bars: 5 μm.

Internalized apoptotic cells are fused with lysosomes where they are further processed [35]. Consistently, in our conditions (MDCK cells infected with PAK), efferocytosed apoptotic material was found within Lysosomal-associated membrane protein 1 (LAMP1) vesicles (S10A Fig). We reasoned that intracellular *P*. *aeruginosa* might have a similar fate. As shown in S10B Fig, PAK-GFP is localized, along with apoptotic debris, within LAMP1 vesicles as well. We then analyzed the presence of LAMP1 in *P*. *aeruginosa*-containing vesicles at different times after internalization. We found more than 80% of intracellular *P*. *aeruginosa* within LAMP1-positive vesicles over time (Fig 6A & 6B and S1 Movie). As functional mature lysosomes are characterized by a low pH, infected cells were stained with LysoTracker, a fluorescent dye that preferentially accumulates in vesicles with an acidic pH. We found that between 45 and 70% intracellular *P*. *aeruginosa* was within acidic vesicles (Fig 6C & 6D), suggesting that its intracellular fate was mature lysosomes. We would have expected the percentage of bacteria inhabiting acidic vesicles to increase through time. However, it is important to consider that only bacteria with some degree of integrity would be visualized with this technique. Severely damaged bacteria would probably lose the GFP label.

Many harmful microorganisms are destroyed inside lysosomes. Thus, we then evaluated intracellular *P*. *aeruginosa* survival inside epithelial cells (Fig 6E). MDCK monolayers were incubated with *P*. *aeruginosa* (MOI = 10) for 2 h. After killing of extracellular bacteria with 400 μg/ml of Amikacin plus 100 μg/ml of Carbenicillin, intracellular *P*. *aeruginosa* survival was monitored at 3, 6, 9 and 24 h post infection in the presence of 40 μg/ml of Amikacin. Extracellular contamination was controlled by CFU counting in the culture. A live-dead staining was carried out to assure that the antibiotics treatment killed most surface-attached bacteria (S11 Fig). General viability of epithelial cells was unchanged throughout the experiment as assessed by trypan blue exclusion (S12A Fig). Also, epithelial cells with internalized bacteria were consistently found inserted in the monolayer. We performed control experiments where Annexin V staining was carried out at 3, 6 and 9 h after infection, and we found (Chi square test) that cells with internalized bacteria and cells with apical Annexin V staining were independent variables (S12B Fig). This indicates that epithelial cells were, at least, not severely damaged by the presence of intracellular bacteria.

The number of intracellular bacteria increased from 3 to 6 h post-infection, then decreased at 9 h, and reached zero at 24 h (Fig 6E). A similar intracellular survival pattern was found for cystic fibrosis isolate 2b (S13 Fig). *P*. *aeruginosa* inhabits LAMP1 vesicles in 16HBE14o- cells as well (S14 Fig). As seen in MDCK cells, bacteria did not survive inside these bronchial epithelial cells beyond 24 h (S15 Fig).

Our results indicate that *P*. *aeruginosa* is eliminated inside epithelial cells, presumably by the lysosomal machinery.

## Discussion

In the present study, we showed that *P*. *aeruginosa* adheres to apoptotic cells extruded from the epithelial barrier and is then internalized through efferocytosis by surrounding epithelial cells. As mentioned, a polarized epithelium provides a sufficient barrier to prevent *P*. *aeruginosa* infection. In healthy individuals, apoptotic cells are quickly removed and disposed of. However, contexts that favor the presence of dead cells might offer an opportunity for *P*. *aeruginosa* colonization.

CF is characterized by exaggerated inflammation, progressive tissue damage, and bacterial colonization, mainly in the respiratory tract. As mentioned, chronic lung infection with *P*. *aeruginosa* is the cause of much of the morbidity and most of the mortality associated with this disease. CF patients typically show an exacerbated number of apoptotic cells in the airways [36]. Although the reasons are not completely clear, cystic fibrosis transmembrane conductance regulator (CFTR)-deficient epithelial cells seem to be more susceptible to apoptosis than cells expressing wild type CFTR [36]. In addition, CF is associated with ineffective efferocytosis of apoptotic cells by either professional phagocytes [4] or neighboring epithelial cells [5]. According to our results, exacerbated apoptosis as well as failed efferocytosis could strengthen infection by promoting *P*. *aeruginosa* colonization and preventing bacterial killing respectively. Therefore, modulating efferocytosis, which may be amenable for pharmacological targeting, could be an attractive therapeutic approach that besides reducing inflammation may help specifically to control *P*. *aeruginosa* infection.

Our findings could also be relevant for the study of *P*. *aeruginosa* infections in the setting of wounds and burns. It is well known that *P*. *aeruginosa* preferentially adheres to and colonizes damaged epithelia [37, 38]. This has often been explained by *P*. *aeruginosa* propensity to adhere to exposed basolateral surfaces or to non-polarized migrating cells at the leading edge. However, wounds and burns are also characterized by the presence of both necrotic and apoptotic cells. Thus, the dead cell tropism of *P*. *aeruginosa* could be another important factor contributing to colonization of damaged epithelia. While we were preparing this manuscript, Schwarzer et al. reported that *P*. *aeruginosa* strain PA01 migrates to dying cells in scratch-wounded epithelia and that some bacteria remain immobilized on dead cells [39].

The intersection of efferocytosis and microbial pathogens has been described for a few examples. All represent cases of microbes inhabiting inside an immune cell that undergoes apoptosis and is engulfed by a phagocyte. Two possible outputs have been observed: 1) The pathogen is eliminated along with the internalized apoptotic cell. An example of this is *Mycobacterium tuberculosis*. This bacterium mostly induces necrotic death of the macrophage it infects. However, some *M*. *tuberculosis*-infected macrophages die by apoptosis, which has long been associated with inhibition of bacterial growth [40, 41]. These apoptotic infected macrophages are rapidly engulfed by uninfected macrophages into which *M*. *tuberculosis* is killed by lysosomes [42]. Similarly, efferocytosis of *Mycobacterium marinum*-infected macrophages by uninfected neutrophils leads to bacterial killing by oxidative mechanisms [43]. 2) The pathogen uses the process of efferocytosis to reach a different cellular host. An example of this is *Leishmania major*. The infectious sand fly bite recruits neutrophils that rapidly phagocytose *L*. *major* metacyclic promastigotes. Parasite infection induces neutrophil apoptosis. *In vitro* models have shown that these infected apoptotic neutrophils are efferocytosed by macrophages, where *L*. *major* survives and replicates [44]. Similarly, *Yersinia pestis*-infected neutrophils are efferocytosed by macrophages, where *Y*. *pestis* also survives and replicates [45]. The case described in the present work would fit into the first output, but provides some remarkable novel features to the emerging efferocytosis-microbial pathogen field. First, the pathogen is not internalized but attached to apoptotic cells. Second, the efferocyte is a non-professional phagocyte.

The presence of *P*. *aeruginosa* might be further engaging the machinery implicated in dead cell removal. In this regard, there is growing evidence showing that opsonins and receptors involved in the removal of pathogens can also aid in the disposal of dying cells, particularly plasma membrane damaged cells such as late apoptotic and necrotic cells (reviewed in [46]). Besides, some signal transducer enzymes, such as PI3K, play a critical role in both efferocytosis and pathogen entry. It has been described that the PI3K product PtdIns(3,4,5)P3 is transiently enriched at the phagocytic cup of macrophages during efferocytosis [31]. We have reported that those membrane protrusions formed at sites where *P*. *aeruginosa* aggregates are enriched in PtdIns(3,4,5)P3, that phosphorylation of the PI3K downstream effector Akt is increased by *P*. *aeruginosa* infection and that PI3K activation is critical for *P*. *aeruginosa* internalization into epithelial cells [14, 47].

As mentioned, Fleiszig’s group has reported that *P*. *aeruginosa* can survive intracellularly into bacteria-induced membrane blebs [20]. These authors further showed that this phenomenon is ExoS-dependent as Type Three Secretion System and ExoS mutants are unable to induce blebs and are instead transported to LAMP3-positive [20] acidic perinuclear vesicles [21]. Our results show that wt *P*. *aeruginosa* enters polarized epithelial cells associated to an “apoptotic cell carrier” along with which it is then transported to acidic LAMP1-positive vesicles. We presume that by this route *P*. *aeruginosa* enters epithelial cells rather passively. Different studies have shown that *P*. *aeruginosa* internalization requires interaction with host cell targets such as lipid rafts [48, 49], caveolin-1 [50] and -2 [51] or CFTR [50, 52]. Also, it has been shown that entry involves the actin [53, 54] and microtubules [55] cytoskeleton as well as host cell signaling proteins such as Src family of tyrosine kinases [56, 57], MER-ERK [58] and Abl tyrosine kinase [59]. Whether these factors are specifically involved in other routes of *P*. *aeruginosa* internalization or they are also part of the entry mechanism described in this work, will need to be tested by future research.

While we cannot rule out that a subpopulation of bacteria escape at some point from inside epithelial cells to the extracellular milieu, our findings highlight the ability of epithelial cells to eliminate *P*. *aeruginosa* through efferocytosis. Thus, we speculate that this could be part of a host defense mechanism. Very recently, a paper showing that *P*. *aeruginosa* transmigrates through a paracellular route at epithelial cell-cell junctions at sites of cell division and dying cell extrusion came out [60]. The authors speculate that this could be a way to achieve systemic infection. Although we have not seen transmigration with our settings, we think that overall these two studies evidence the complexity of the *P*. *aeruginosa*-epithelial barrier interplay. It can be speculated that as *P*. *aeruginosa* arrives to sites of apoptotic cell extrusion, bacterial pathogenic as well as host defense mechanisms come into play: Thus, bacteria could transmigrate, and reach deeper tissues or it could be eliminated through efferocytosis upon binding to apoptotic cells.

We also speculate that apoptotic cells or apoptotic bodies with *P*. *aeruginosa* associated to their surface could be very attractive targets for professional phagocytes as well. Beyond direct killing of bacteria, acquiring apoptotic material containing bacterial antigens might play a role in stimulation of the adaptive immune response. It has been shown that infected macrophages that have undergone apoptosis can be uptaken by dendritic cells which in turn present epitopes from pathogen-derived antigens on either MHC-I or both MHC-I and MHC-II [61, 62]. In the future, it will be of interest to investigate the implications of the dead cell tropism of *P*. *aeruginosa* in both innate and adaptive immune responses.

As mentioned, we hypothesize that elimination of *P*. *aeruginosa* through efferocytosis might be part of a host defense mechanism. In healthy individuals, apoptotic cells are rapidly cleared. In this context, *P*. *aeruginosa* will have reduced chances to attach, and if it does, it will be quickly eliminated. However, in contexts where apoptotic cells are unusually produced and/or efferocytosis fails, *P*. *aeruginosa* will have an opportunity for colonization.

## Materials & Methods

### Antibodies and reagents

Anti-*P*. *aeruginosa* (ab68538) and anti-LAMP1 (ab24170) antibodies were obtained from AbCam. LysoTracker-DND99 Red, Alexa conjugated Annexin V, Rhodamine and Alexa-fluor 647 conjugated phalloidin, TO-PRO3 and CellTrace were acquired from Life Technologies. Cleaved Caspase-3 antibody (9661S) was obtained from Cell Signaling Technology. Purified Recombinant Annexin V was either purchased from BD Pharmingen or produced in the laboratory from pProEx.Htb.Annexin V plasmid (kindly provided by Dr. Seamus J. Martin)[63].

### Cell preparation and culture

Wt MDCK (clone II, generously gifted by Dr. Keith Mostov), Lifeact-GFP MDCK (generously gifted by Drs. Liang Cai and Keith Mostov), MDCK Rac1-N17 (generously gifted by Dr. James Nelson) and 16HBE14o- cells (generously gifted by Dr. Alan Verkman) were cultured in MEM containing 5% (wtMDCK and Lifeact-GFP MDCK) or 10% fetal bovine serum (MDCK Rac1-N17 and 16HBE14o-) at 37°C with 5% CO_2_. G418 (500 μg/ml) was added to MDCK Rac1-N17 culture media. MDKC cells were seeded on a 12-mm-diameter transwell (Corning Fisher, NY, USA, 4.5×10^5^ cells per transwell) and used for experiments after 48 h in culture. When grown on glass coverslips, cells were seeded at high density (0.5×10^5^ cells x cm^-2^) and grown for 4 days without changing the medium. 16HBE14o- cells were seeded on transwells (1×10^5^ cells per 12 mm diameter transwell) and used for experiments after 7 days in culture.

### Bacterial infection

Three *P*. *aeruginosa* strains were used in this study: *P*. *aeruginosa* strain K (PAK), and isolates 2b and 6 [28], from two different cystic fibrosis patients (generously provided by Dr. Andrea Smania, Universidad Nacional de Córdoba, Argentina).

*Pseudomonas aeruginosa* was routinely grown shaking overnight in Luria-Bertani broth at 37°C. For fluorescence microscopy studies, *P*. *aeruginosa* carrying plasmids containing either the mCherry (pMP7605 plasmid was generously gifted by Dr. Lagendijk [64]) or GFP [65] genes was used. Stationary-phase bacteria were co-incubated with epithelial cells at a MOI of 60 unless otherwise indicated. Standard internalization assays were performed as described previously [66].

### Microscopy studies

Transwell or coverslip grown cells were infected with *P*. *aeruginosa* for the indicated times and washed with PBS. Samples were fixed with 4% paraformaldehyde in PBS for 15 min at room temperature, blocked with fish skin gelatin 0.7%, permeabilized with saponin 0.1% and then incubated overnight at 4°C with the primary antibody and for 1 h at 37°C with the secondary antibody. Samples were stained with TO-PRO3 for 15 min and with phalloidin for 60 min. Annexin V, LysoTracker and Propidium Iodide staining were performed on live samples [67]. For Annexin V staining, samples were washed with binding buffer (BB): 10 mM HEPES, 140 mM NaCl and 2.5 mM CaCl2, pH 7.4, and incubated with Alexa conjugated-Annexin V (1/20 dilution) for 15 min at room temperature. Samples were then fixed with 4% paraformaldehyde in BB, permeabilized with saponin 0.025% in BB (permeabilization buffer) for 15 min, blocked with BSA 1% in permeabilization buffer for 60 min.

To distinguish live from dead bacteria, samples were stained with the BacLight Bacterial Viability Kit (L7007, ThermoFisher) according to the manufacturer’s instructions.

Samples were examined with a confocal laser-scanning microscope Olympus FV1000 using a PlanApo N (60X 1.42 NA) oil objective.

For electron microscopy studies, samples were loaded on carbon-coated copper grids, negatively stained with 1% uranyl acetate for 2 min at room temperature, and imaged using a JEOL (Peabody, MA, USA) JEM 1010 electron microscope operated at 80 kV.

### Apoptotic and necrotic cells

To make apoptotic cells, confluent MDCK or 16HBE14o- cells were trypsinized, resuspended in serum-free MEM, plated and treated with short-wave UV (6 mj/cm^2^) in a CL-100 Ultraviolet Crosslinker, UVP, for 2 min. Cells were then incubated at 37°C in 5% CO_2_ for 12 h. Alternatively supernatants containing dead cells from overgrown MDCK monolayers were used [11]. To make necrotic cells, MDCK cells were exposed to 100 mM H_2_O_2_ for 30 min and incubated at 37°C in 5% CO_2_ for 20 h in serum-free MEM [68].

### Addition of apoptotic or necrotic cells to monolayers

Apoptotic or necrotic wt MDCK cells were labeled with Alexa conjugated Annexin in BB for 15 min, washed and resuspended in serum-free MEM. The labeled cells were then added to confluent MDCK monolayers (5 added cells/100 cells in the monolayer) and incubated for the indicated times at 37°C in 5% CO_2_.

### Efferocytosis inhibition

Transwell grown monolayers were washed three times with BB and incubated with BB (control) or unlabeled recombinant Annexin V (15 μg/10^5^ cells) for 15 min at room temperature. Cells were incubated back in serum-free MEM and infected with PAK-mCherry. Extracellular *P*. *aeruginosa* was labeled with anti-*P*. *aeruginosa* antibody. Intracellular/extracellular bacteria were quantified as previously described [34]. In a different set of experiments, apoptotic cells isolated from supernatants of overgrown MDCK cultures were incubated with unlabeled recombinant Annexin V (15 μg/10^5^ cells) or with BB alone (control) for 15 min at room temperature. Apoptotic cells were then added to monolayers and infection was carried out right after. Standard internalization assays were performed.

### Image analysis

Images were analyzed using the Image J program (National Institutes of Health, NIH, USA). To quantify the monolayer-associated bacteria and monolayer-associated apoptotic cells/apoptotic bodies, we used the 3D-Object Counter plugin for ImageJ. This plugin identifies and enumerates the objects in the stack with a user-defined threshold for voxel intensity value. Then, the plugin generates a binary mask showing the particles and provides a list of the particles with their respective volumes (S7 Fig). Localization of the particles (i.e. intracellular or extracellular) was determined visually. When indicated, bacterial localization was determined by staining non-permeabilized samples with an anti-*Pseudomonas* antibody, as described in [34].

When indicated, fluorescence microscopy confocal image stacks were projected along the z-axis, creating an output image in which each pixel contained the maximum intensity pixel value in that particular x-y position.

### Statistical analysis

Data are shown as mean ± standard error of the mean (SEM) of at least three independent experiments. Student´s t-test, One sample t-test, Pearson Correlation or Chi square test were applied using GraphPad Prism version 6.01. A *p* value < 0.05 was considered statistically significant.

## Supporting Information

S1 Fig Cystic fibrosis isolates of *P*. *aeruginosa* aggregate on extruded apoptotic cells. (A) Projected confocal Z stacks of transwell-grown MDCK monolayers infected with *P*. *aeruginosa* cystic fibrosis isolates. Extruded apoptotic cells were visualized with Annexin V staining (green). Strain 6 was labeled with anti-*Pseudomonas* antibody (red), and strain 2b expressed mCherry (red). Phalloidin: blue. Scale bar: 10 μm. (B) Percentage of aggregates formed at Annexin V-positive sites.
(PDF)

S2 Fig *P*. *aeruginosa* aggregates on extruded 16HBE14o- apoptotic cells. (A) 16HBE14o- human bronchial epithelial cell layers infected with PAK-mCherry (red) and stained with Annexin V-Alexa 488 (green) and phalloidin (blue). Scale bar: 10 μm. (B) Percentage of aggregates formed at Annexin V-positive sites.
(PDF)

S3 Fig Generated apoptotic and necrotic cells. Late apoptotic (left) and necrotic (right) MDCK cells generated by UV and H_2_O_2_ respectively. Cells were stained with Annexin V-Alexa 488 (green) and nuclei with Propidium Iodide (red). Scale bars: 10 μm.
(PDF)

S4 Fig *P*. *aeruginosa* preferentially adheres to dead over live cells. UV generated apoptotic wtMDCK cells were mixed with trypsin-detached Lifeact-GFP MDCK cells, stained with Annexin V-Alexa 647 and added to glass-grown wtMDCK monolayers followed by PAK-mCherry infection and incubation for 3h. Projected confocal Z stack shows that PAK (red) preferentially adheres to dead cells (blue) over living cells (green). Scale bar 20 μm.
(PDF)

S5 Fig Efferocytosis takes place in cultured MDCK monolayers. Lifeact-GFP MDCK monolayers (green) were stained with Annexin V-Alexa 647 (blue) and incubated for 3 h. Confocal xy plane (top) and orthogonal section (bottom) showing an efferocytic phagosome. Scale bar: 5 μm.
(PDF)

S6 Fig Internalized cystic fibrosis isolates are inside cells that also have intracellular apoptotic cell debris. (A) Extruded apoptotic cells in transwell-grown MDCK monolayers were labeled with fluorescent Annexin V (green). Monolayers were then infected with the cystic fibrosis isolates. Strain 2b is shown (red). Epithelial cells are visualized by Phalloidin staining (blue). Scale bar: 10 μm. (B) Percentage of internalized bacteria in cells that also have intracellular apoptotic cell debris.
(PDF)

S7 Fig *P*. *aeruginosa* internalizes into 16HBE14o- cells through efferocytosis. 16HBE14o- layers were stained with Annexin V-Alexa 488 (green), infected with PAK-mCherry (red) and incubated for 3 h. Samples were fixed and stained with phalloidin for F-actin (blue). Confocal xy plane (top) and orthogonal section (bottom) showing an intracellular vesicle containing both apoptotic cell debris and bacteria. Scale bar: 5 μm.
(PDF)

S8 Fig Representative image showing how the Object counter tool from ImageJ is used to evaluate the volume of monolayer-associated apoptotic cell material. (A) CellTrace (blue) labeled apoptotic cells associated to lifeact-GFP monolayers (green). (B) “Object or particle map” rendered by the Object counter tool. (C) Chart listing the volume (in voxels) of the particles. The localization (i.e. extracellular or intracellular) of apoptotic material was defined visually.
(PDF)

S9 Fig Total monolayer-associated bacteria after pre-incubation with AnnexinV. Proportion of total monolayer-associated *P*. *aeruginosa* after pre-incubating transwell-grown lifeact-GFP MDCK monolayers with unlabeled Annexin V for 15 min in binding buffer or with binding buffer alone (control). Data were normalized to control. NS: not significant.
(PDF)

S10 Fig Internalized apoptotic material is localized into LAMP1 vesicles. Transwell-grown MDCK monolayers were stained with Annexin V-Alexa 647 (blue), infected either with wtPAK (A) or PAK-GFP (B) and incubated for 3 h. (A) XY plane showing a LAMP1-positive vesicle containing apoptotic material. F-actin: red, LAMP1: green. (B) XY plane showing a LAMP1-positive vesicle containing apoptotic material and bacteria. PAK-GFP: green, LAMP1: red. Scale bars: 5 μm.
(PDF)

S11 Fig Antibiotics treatment kills surface-aggregated bacteria. Live imaging of MDCK monolayers infected with PAK. Bacterial viability after exposure to Amikacin plus Carbenicillin was evaluated by staining live bacteria with SYTO 9 (green) and counterstaining dead bacteria with propidium iodide (red).
(PDF)

S12 Fig Epithelial cell viability. (A) Viability of MDCK cells throughout the intracellular PAK survival curve was assayed by trypan blue exclusion (B) Annexin V staining was carried out at 3, 6 and 9 h after infection of MDCK cells with PAK-GFP (antibiotics were added 2 h after infection as described above). Cells were stained with phalloidin. Number of cells with or without intracellular bacteria and with or without apical Annexin staining was quantified. A Chi square test indicated that cells with internalized bacteria and cells with apical Annexin V staining are independent variables (3h: p = 0.54 NS, 6h p = 0.69 NS, 9h p = 0.83 NS).
(PDF)

S13 Fig Intracellular cystic fibrosis isolate 2b survival curve in MDCK cells. (PDF)

S14 Fig *P*. *aeruginosa* inhabits LAMP1-positive vesicles inside 16HBE14o- cells. 16HBE14o- layers were infected with PAK for 3 h. Projected confocal Z stack (top) and orthogonal section (bottom) showing LAMP1-positive vesicles containing bacteria. F-actin: blue, PAK-GFP: green and LAMP1: red. Scale bar: 5 μm.
(PDF)

S15 Fig Intracellular PAK survival curve in 16HBE14o- cells. (PDF)

S1 Movie *P*. *aeruginosa* inhabits LAMP1-positive vesicles. Complete optical scan of MDCK cells infected with PAK-GFP (green), and stained with phalloidin (blue) and LAMP1 (red) (Fig 6A). Scanning proceeds from the apical to the basolateral surface.
(AVI)
