# Supplementary figures and images for: Elimination of Pseudomonas aeruginosa through Efferocytosis upon Binding to Apoptotic Cells
Source: PLoS Pathog. 2016 Dec 15;12(12):e1006068. doi: 10.1371/journal.ppat.1006068 (PMC5158079; doi:10.1371/journal.ppat.1006068)

**A****Non-mucoid  
Strain 6****Mucoid  
Strain 2B****Annexin V**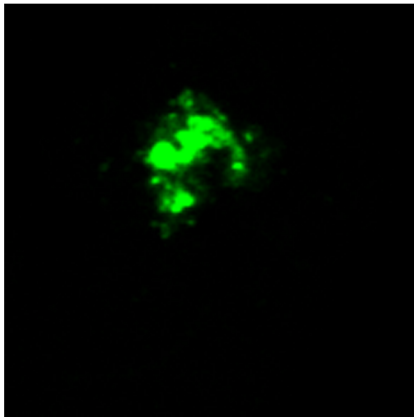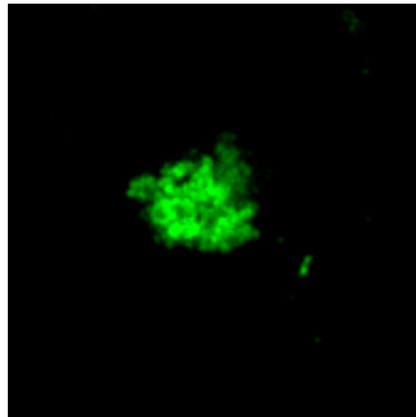***P. aeruginosa***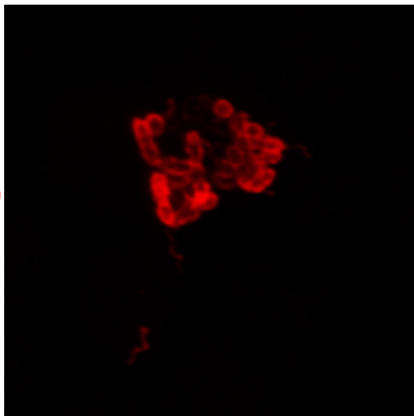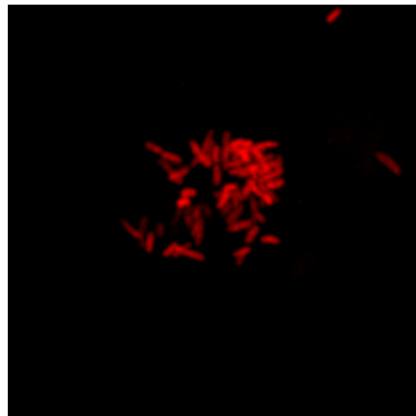**F-Actin**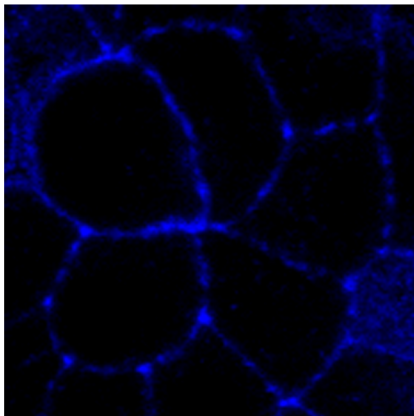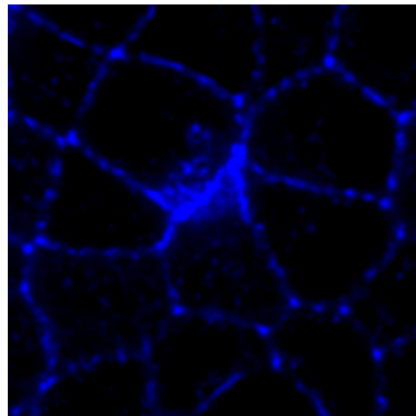**Merge**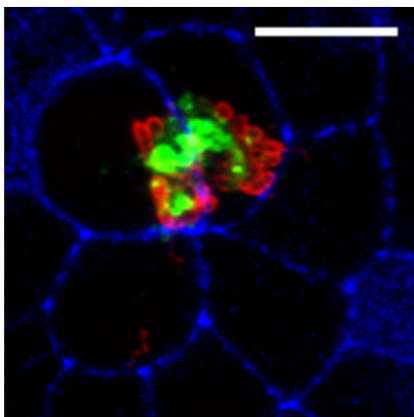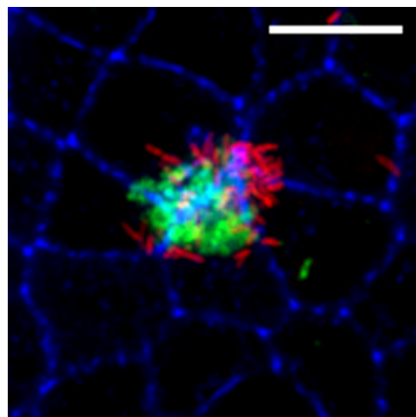**B**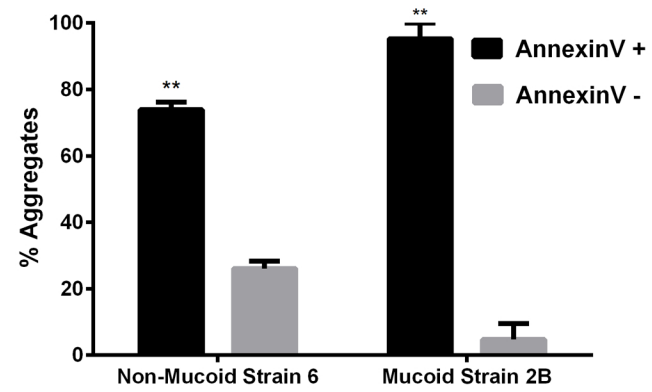

Supplement: S1 Fig — (A) Projected confocal Z stacks of transwell-grown MDCK monolayers infected with P. aeruginosa cystic fibrosis isolates. Extruded apoptotic cells were visualized with Annexin V staining (green). Strain 6 was labeled with anti-Pseudomonas antibody (red), and strain 2b expressed mCherry (red). Phalloidin: blue. Scale bar: 10 μm. (B) Percentage of aggregates formed at Annexin V-positive sites. (PDF) [file ppat.1006068.s001.pdf]

**A****PAK-mCherry**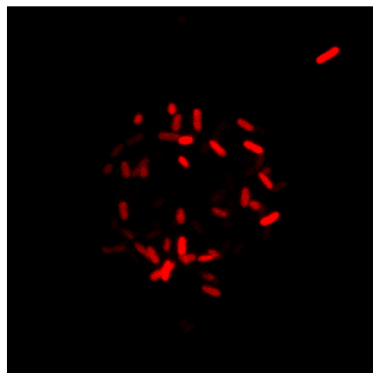**Annexin V**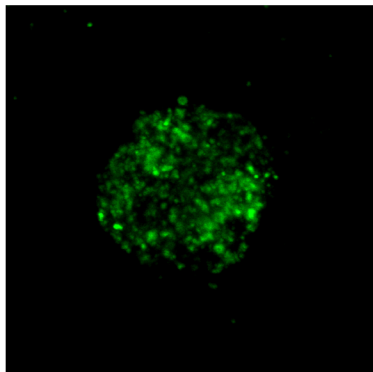**F-Actin**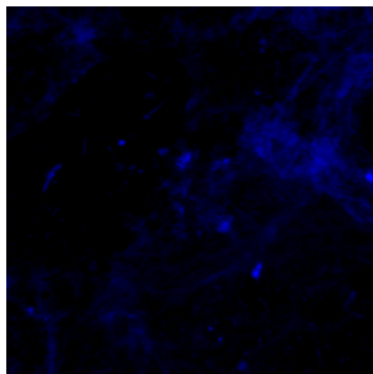**Merge**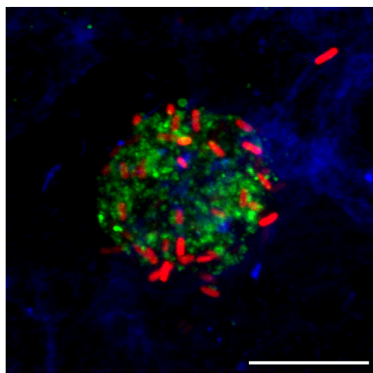**B**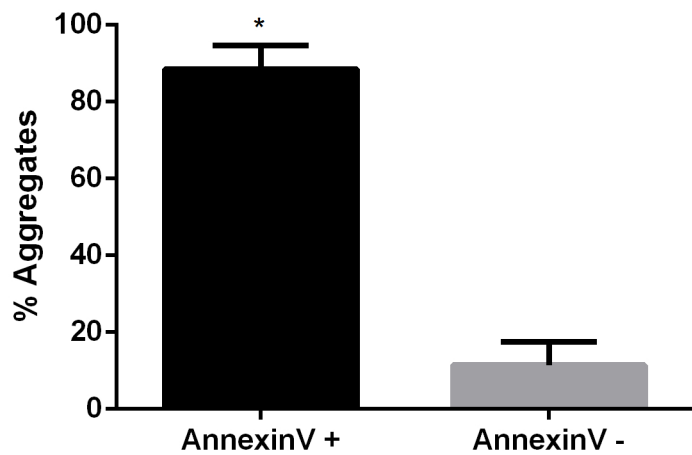

Supplement: S2 Fig — (A) 16HBE14o- human bronchial epithelial cell layers infected with PAK-mCherry (red) and stained with Annexin V-Alexa 488 (green) and phalloidin (blue). Scale bar: 10 μm. (B) Percentage of aggregates formed at Annexin V-positive sites. (PDF) [file ppat.1006068.s002.pdf]

**Apoptotic**

**Necrotic**

Annexin V/PI

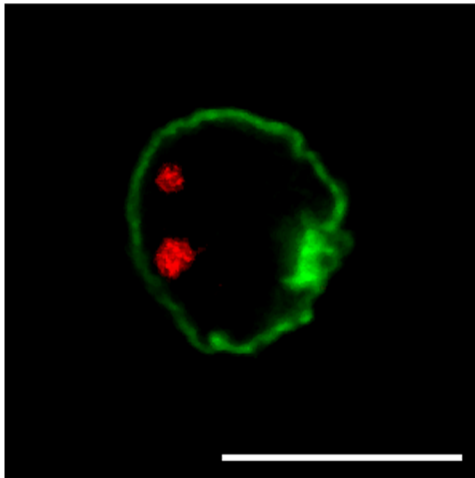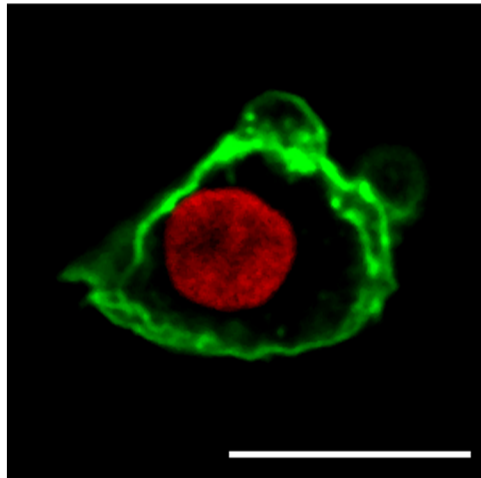

Supplement: S3 Fig — Late apoptotic (left) and necrotic (right) MDCK cells generated by UV and H2O2 respectively. Cells were stained with Annexin V-Alexa 488 (green) and nuclei with Propidium Iodide (red). Scale bars: 10 μm. (PDF) [file ppat.1006068.s003.pdf]

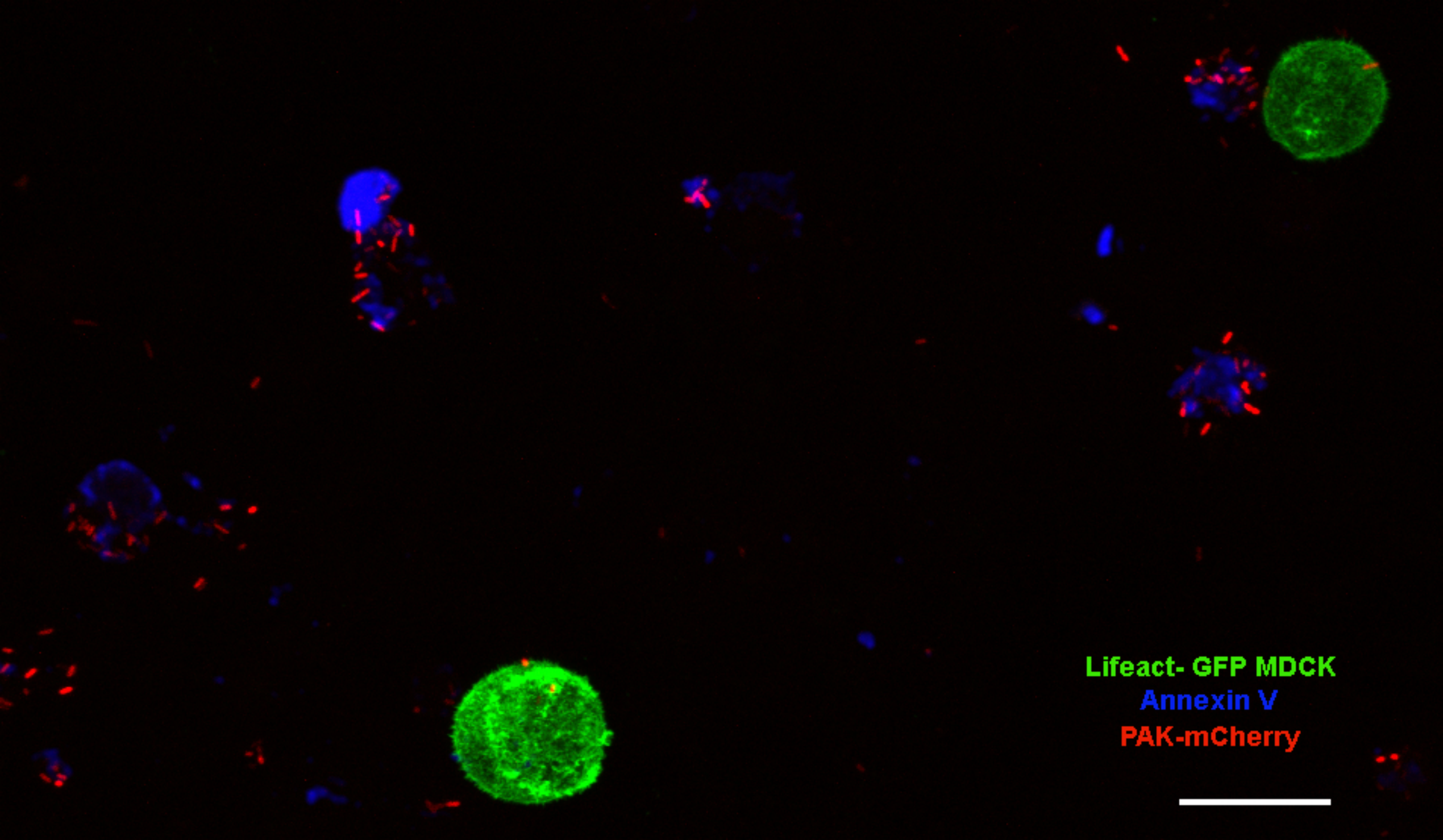

**Lifeact- GFP MDCK**  
**Annexin V**  
**PAK-mCherry**

Supplement: S4 Fig — UV generated apoptotic wtMDCK cells were mixed with trypsin-detached Lifeact-GFP MDCK cells, stained with Annexin V-Alexa 647 and added to glass-grown wtMDCK monolayers followed by PAK-mCherry infection and incubation for 3h. Projected confocal Z stack shows that PAK (red) preferentially adheres to dead cells (blue) over living cells (green). Scale bar 20 μm. (PDF) [file ppat.1006068.s004.pdf]

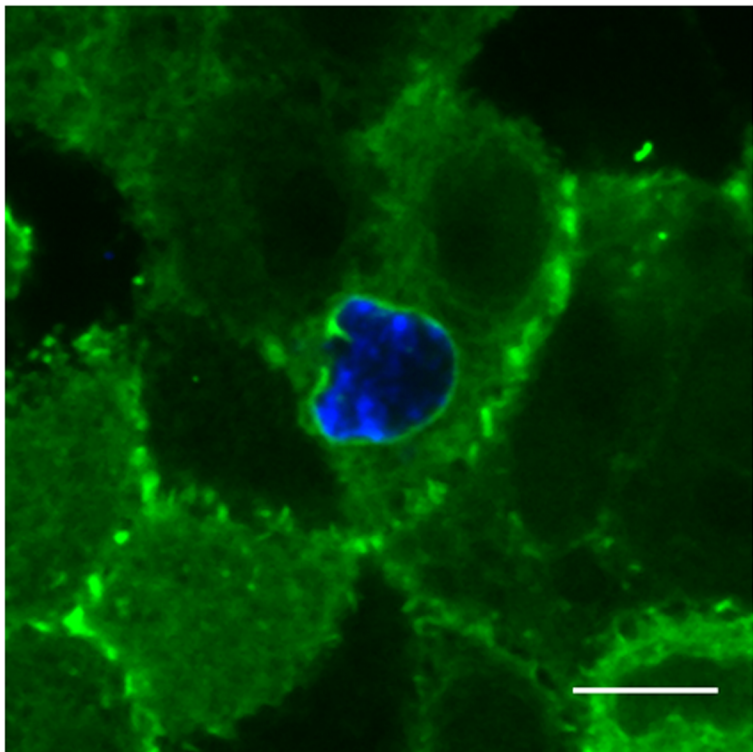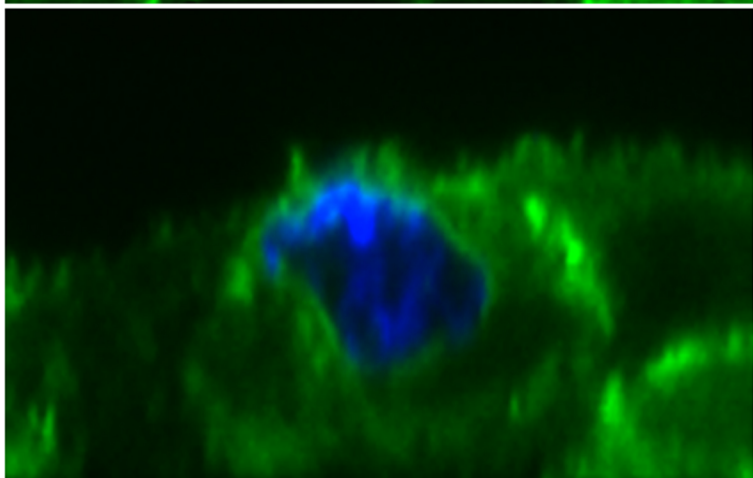

**F-Actin / Annexin V**

Supplement: S5 Fig — Lifeact-GFP MDCK monolayers (green) were stained with Annexin V-Alexa 647 (blue) and incubated for 3 h. Confocal xy plane (top) and orthogonal section (bottom) showing an efferocytic phagosome. Scale bar: 5 μm. (PDF) [file ppat.1006068.s005.pdf]

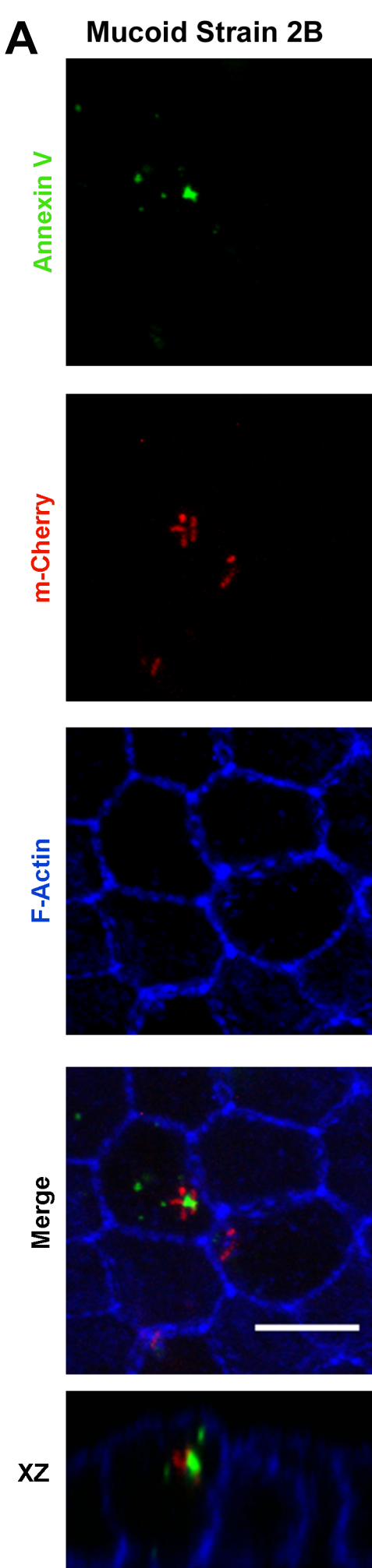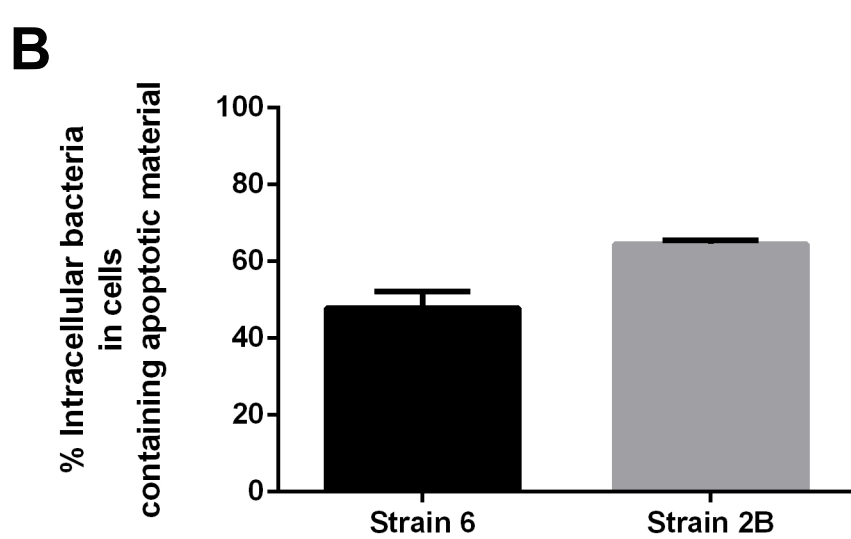

Supplement: S6 Fig — (A) Extruded apoptotic cells in transwell-grown MDCK monolayers were labeled with fluorescent Annexin V (green). Monolayers were then infected with the cystic fibrosis isolates. Strain 2b is shown (red). Epithelial cells are visualized by Phalloidin staining (blue). Scale bar: 10 μm. (B) Percentage of internalized bacteria in cells that also have intracellular apoptotic cell debris. (PDF) [file ppat.1006068.s006.pdf]

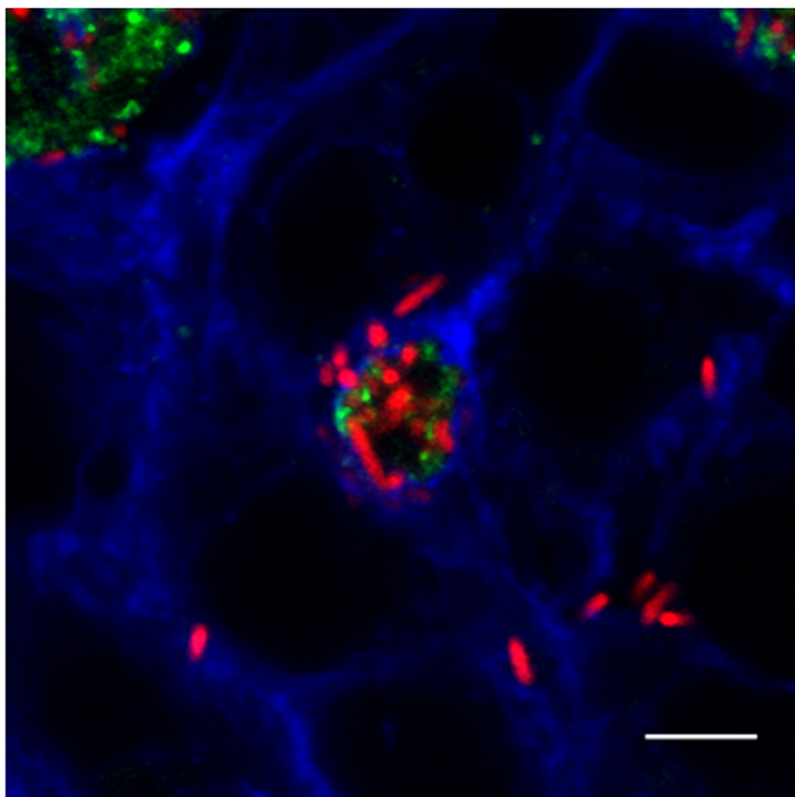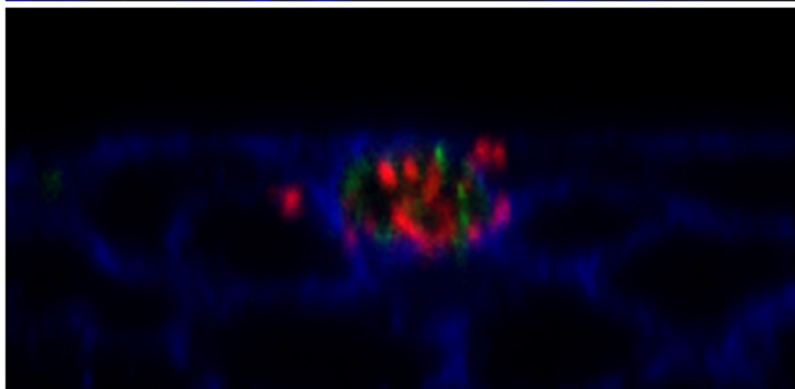

**F-Actin** / **PAK-mCherry** / **Annexin V**

Supplement: S7 Fig — 16HBE14o- layers were stained with Annexin V-Alexa 488 (green), infected with PAK-mCherry (red) and incubated for 3 h. Samples were fixed and stained with phalloidin for F-actin (blue). Confocal xy plane (top) and orthogonal section (bottom) showing an intracellular vesicle containing both apoptotic cell debris and bacteria. Scale bar: 5 μm. (PDF) [file ppat.1006068.s007.pdf]

**A**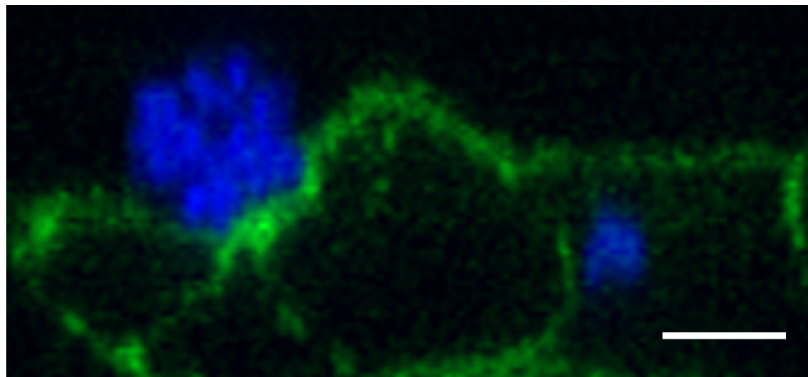

**F-Actin / CellTrace**

**B**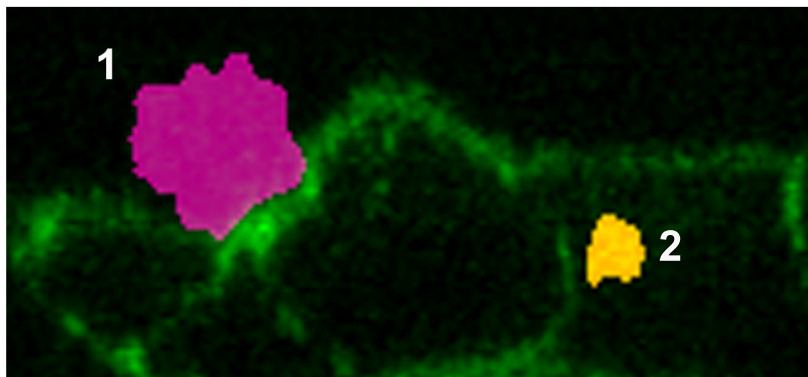**C**

| Particle # | Volume (Vx) | Position      |
|------------|-------------|---------------|
| 1          | 20316       | Extracellular |
| 2          | 1243        | Intracellular |

Supplement: S8 Fig — (A) CellTrace (blue) labeled apoptotic cells associated to lifeact-GFP monolayers (green). (B) “Object or particle map” rendered by the Object counter tool. (C) Chart listing the volume (in voxels) of the particles. The localization (i.e. extracellular or intracellular) of apoptotic material was defined visually. (PDF) [file ppat.1006068.s008.pdf]

**Monolayer-associated  
Bacteria**

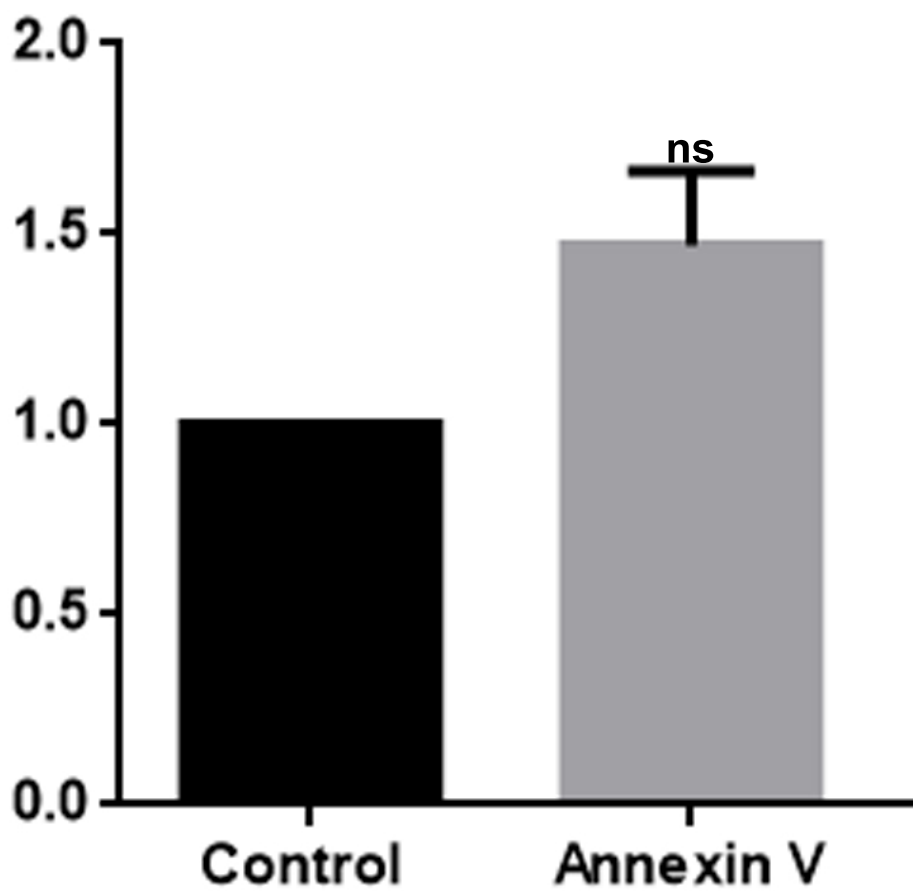

Supplement: S9 Fig — Proportion of total monolayer-associated P. aeruginosa after pre-incubating transwell-grown lifeact-GFP MDCK monolayers with unlabeled Annexin V for 15 min in binding buffer or with binding buffer alone (control). Data were normalized to control. NS: not significant. (PDF) [file ppat.1006068.s009.pdf]

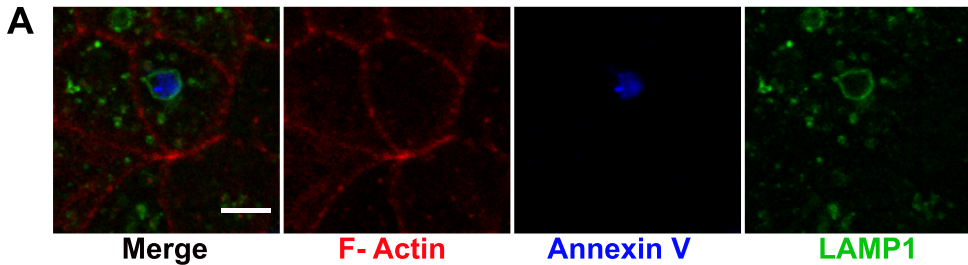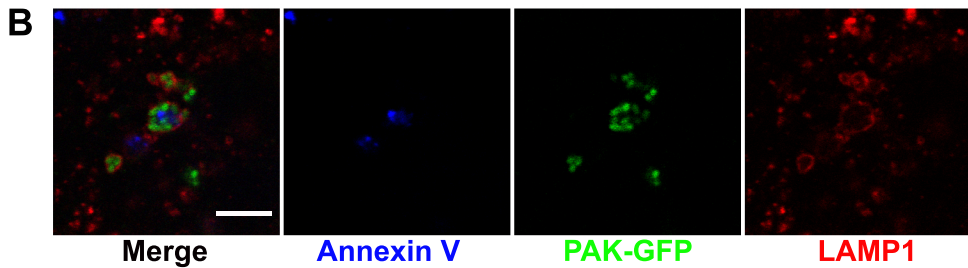

Supplement: S10 Fig — Transwell-grown MDCK monolayers were stained with Annexin V-Alexa 647 (blue), infected either with wtPAK (A) or PAK-GFP (B) and incubated for 3 h. (A) XY plane showing a LAMP1-positive vesicle containing apoptotic material. F-actin: red, LAMP1: green. (B) XY plane showing a LAMP1-positive vesicle containing apoptotic material and bacteria. PAK-GFP: green, LAMP1: red. Scale bars: 5 μm. (PDF) [file ppat.1006068.s010.pdf]

**A**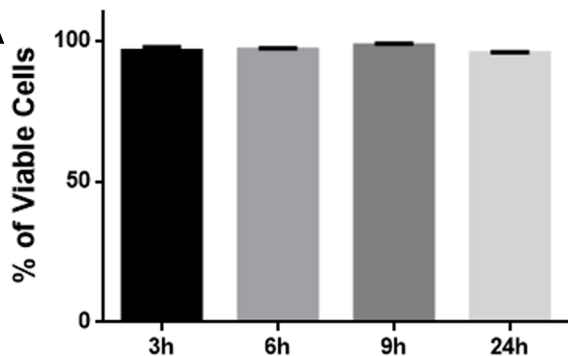**B**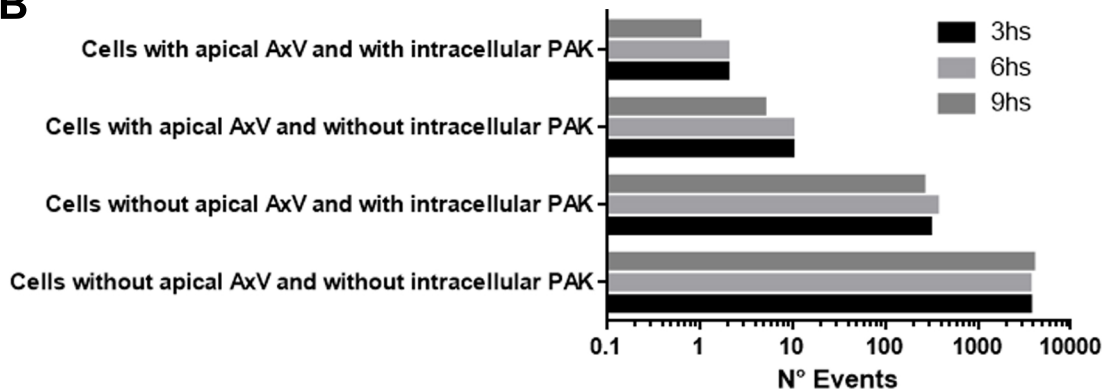

Supplement: S12 Fig — (A) Viability of MDCK cells throughout the intracellular PAK survival curve was assayed by trypan blue exclusion (B) Annexin V staining was carried out at 3, 6 and 9 h after infection of MDCK cells with PAK-GFP (antibiotics were added 2 h after infection as described above). Cells were stained with phalloidin. Number of cells with or without intracellular bacteria and with or without apical Annexin staining was quantified. A Chi square test indicated that cells with internalized bacteria and cells with apical Annexin V staining are independent variables (3h: p = 0.54 NS, 6h p = 0.69 NS, 9h p = 0.83 NS). (PDF) [file ppat.1006068.s012.pdf]

## Mucoid Strain 2B

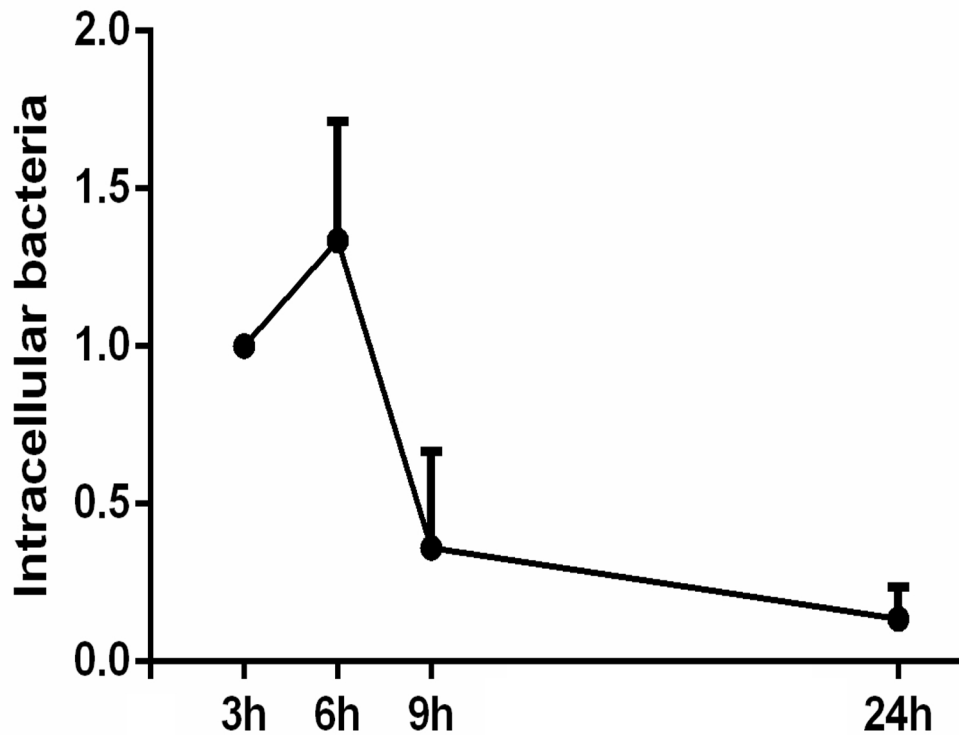

Supplement: S13 Fig — (PDF) [file ppat.1006068.s013.pdf]

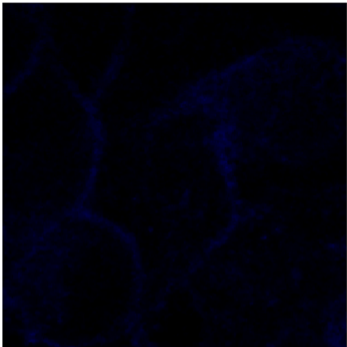

**F-Actin**

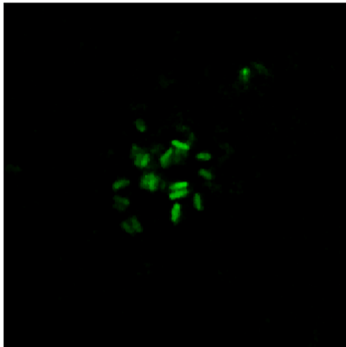

**PAK-GFP**

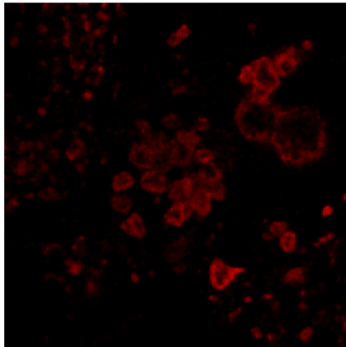

**LAMP1**

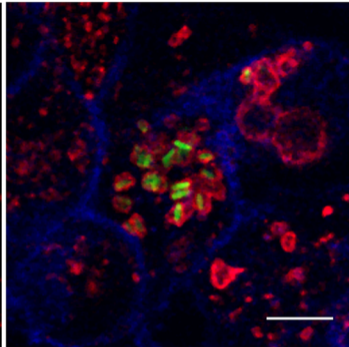

**Merge**

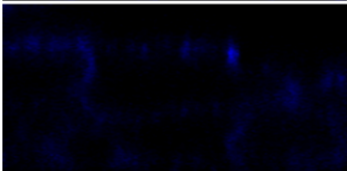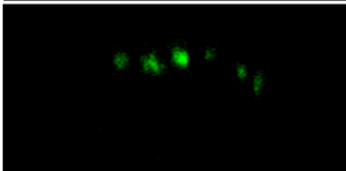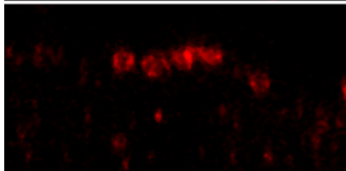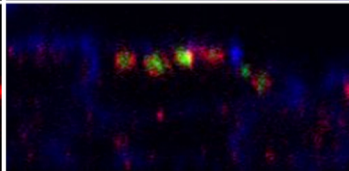

Supplement: S14 Fig — 16HBE14o- layers were infected with PAK for 3 h. Projected confocal Z stack (top) and orthogonal section (bottom) showing LAMP1-positive vesicles containing bacteria. F-actin: blue, PAK-GFP: green and LAMP1: red. Scale bar: 5 μm. (PDF) [file ppat.1006068.s014.pdf]

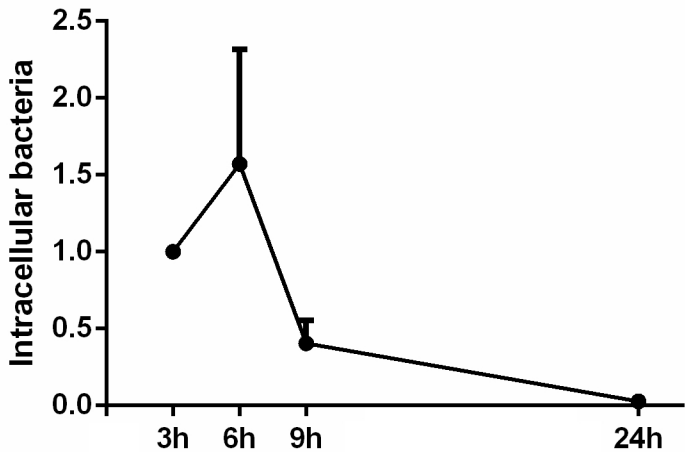

Supplement: S15 Fig — (PDF) [file ppat.1006068.s015.pdf]
