# Supplementary material for: Elimination of Pseudomonas aeruginosa through Efferocytosis upon Binding to Apoptotic Cells
Source: PLoS Pathog. 2016 Dec 15;12(12):e1006068. doi: 10.1371/journal.ppat.1006068 (PMC5158079; doi:10.1371/journal.ppat.1006068)
Supplement: S11 Fig — Live imaging of MDCK monolayers infected with PAK. Bacterial viability after exposure to Amikacin plus Carbenicillin was evaluated by staining live bacteria with SYTO 9 (green) and counterstaining dead bacteria with propidium iodide (red). (PDF) [file ppat.1006068.s011.pdf]

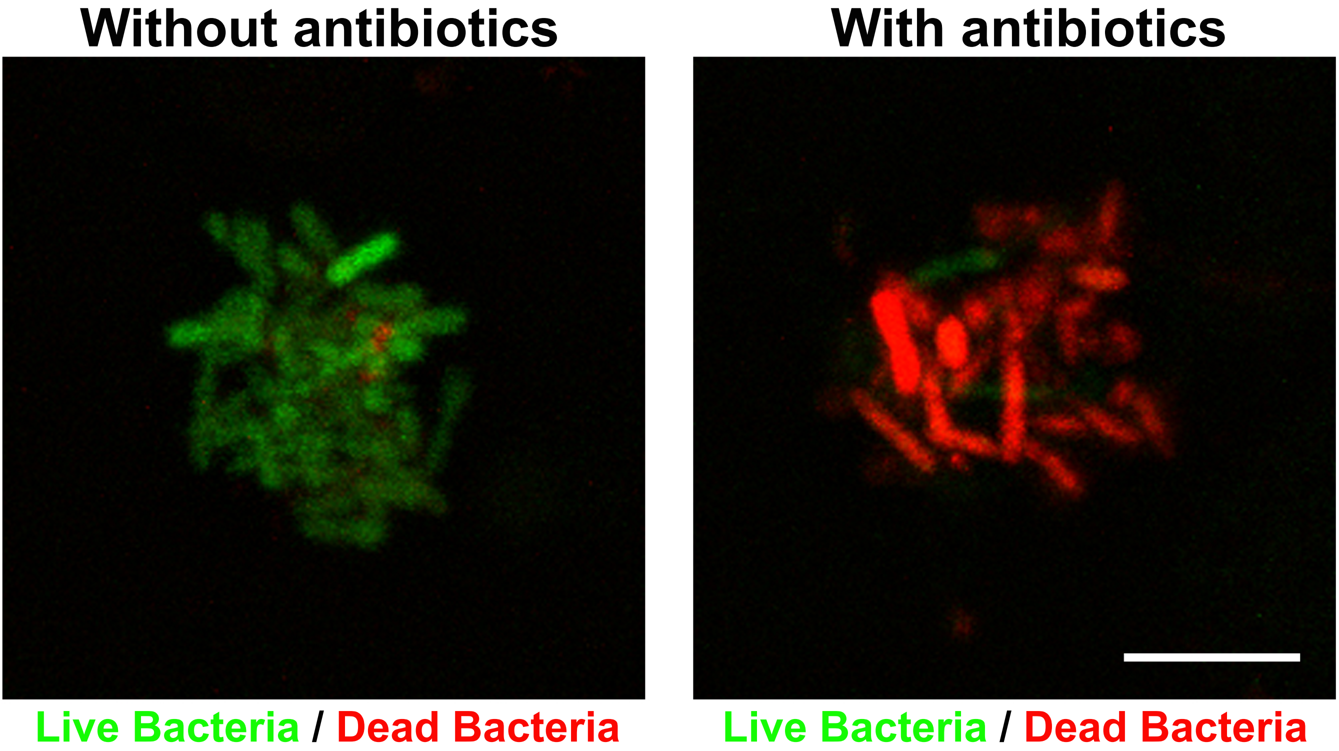
Without antibiotics
With antibiotics
Live Bacteria / Dead Bacteria
Live Bacteria / Dead Bacteria
